# Design, synthesis and response surface optimization of a high-performance LaFeO_3_/Mg–Al layered double hydroxide/chitosan ternary nanocomposite as a pH-responsive carrier for controlled celecoxib release: experimental evaluation and kinetic modeling

**DOI:** 10.1039/d5ra09708h

**Published:** 2026-06-03

**Authors:** Samira Moradi Kozaraji, Seyed Mahdi Mousavi, Moslem Setoodehkhah

**Affiliations:** a Department of Applied Chemistry, Faculty of Chemistry, University of Kashan Kashan Iran mousavi.smahdi@kashanu.ac.ir; b Department of Inorganic Chemistry, Faculty of Chemistry, University of Kashan Kashan Iran

## Abstract

In this study, an advanced ternary nanocomposite, LaFeO_3_/Mg–Al LDH/chitosan, was meticulously designed and synthesized as a pH-responsive controlled drug delivery system for the anti-inflammatory drug celecoxib. The LaFeO_3_ precursor was synthesized *via* the sol–gel method and Mg–Al LDH was prepared through co-precipitation; the final nanocomposite was fabricated using ultrasonication and reflux techniques. Comprehensive characterization employing FT-IR, XRD, SEM, EDS, elemental mapping, and VSM confirmed the successful formation of a layered polymeric hybrid structure with uniform component distribution, ordered sheet-like morphology, and reduced magnetic properties compared to pristine LaFeO_3_. Drug loading was investigated using the post-loading strategy, optimized *via* Response Surface Methodology (RSM) and Central Composite Design (CCD) by tuning key parameters: pH, temperature, and water : ethanol volumetric ratio. Under optimal conditions (pH = 9.38, temperature = 43.92 °C, water : ethanol ratio = 76.757), the drug loading efficiency (DLE) reached 98.72%, and the drug loading capacity (DLC) attained 43.96%, surpassing the performance of binary nanocomposites. The drug release profile exhibited pH-sensitivity, with faster release at pH 5.8 compared to pH 7.4. A biphasic release pattern was observed, characterized by an initial burst phase followed by sustained release, achieving approximately 90% release after 24 hours. The nanocomposite's swelling behavior was significantly higher under acidic conditions, indicating its pH-responsive behavior. Kinetic modeling revealed an anomalous transport mechanism (*n* = 0.62), controlled by combined molecular diffusion and matrix swelling. With its high loading capacity, controlled release, biocompatible main components, and environmental responsiveness, this nanocomposite emerges as a highly promising platform for pH-responsive controlled drug delivery systems.

## Abstract 2

In this study, an advanced ternary nanocomposite, LaFeO_3_/Mg–Al LDH/chitosan, was meticulously designed and synthesized as a pH-responsive controlled drug delivery system for the anti-inflammatory drug celecoxib.

## Graphical abstract

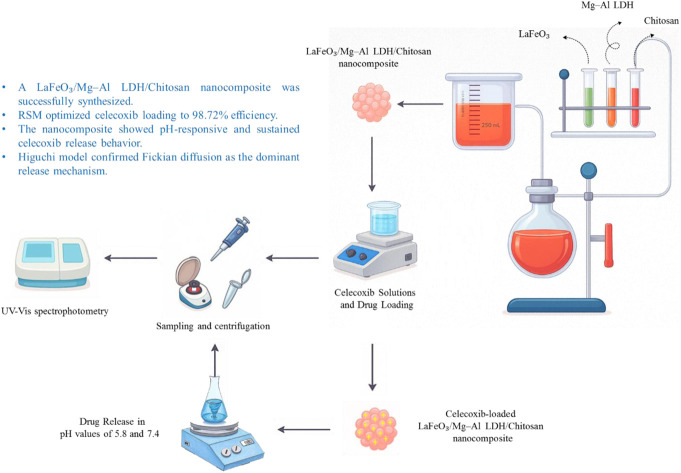

## 1. Introduction

Advanced drug delivery systems are pivotal for precise, controlled release of drugs. Through stimuli-responsive designs based on nanotechnology, they enable targeted release under specific physiological conditions, which maximizes therapeutic efficacy while minimizing side effects. Selective mechanisms, including feedback-driven interactions, deliver the drug directly to the target site, enhancing overall therapeutic efficiency.^1–5^

Nanoparticles are key components in drug delivery.^6^ Their nanoscale size enables efficient cellular penetration, traversal of biological barriers (*e.g.*, blood–brain barrier), and precise molecular interactions. These capabilities help overcome conventional limitations, enabling next-generation therapeutic systems.^7–11^

Integrating nanoparticles into nanocomposites significantly enhances their capabilities.^12^ These structures, composed of diverse materials, offer high drug-loading, improved stability, and stimuli-responsive controlled release.^13,14^ Compared to individual nanoparticles, they provide further advantages like pH-triggered release, protection of sensitive agents, and prolonged drug half-life.^15,16^ By improving therapeutic performance while reducing dosages and side effects, nanocomposites are key to precision medicine.^17^

Polymeric nanoparticles (10–100 nm) are widely used nanocarriers, synthesized from synthetic (*e.g.*, polycaprolactone) or natural (*e.g.*, chitosan) polymers.^18,19^ They can be biodegradable or non-biodegradable.^20^ Biodegradable types hydrolyze into biocompatible, non-toxic monomers, which enhances their clinical safety and efficacy.^21^

Chitosan, a natural polymer, is used extensively in drug delivery due to its biocompatibility, biodegradability, and low cost.^22–25^ Its acidic solubility enables pH-sensitive release systems, and its antioxidant/antimicrobial properties are ideal for antibacterial, anticancer, and gene therapies.^26–28^ Available in various forms (*e.g.*, hydrogels, nanoparticles), its absorbable degradation products make it excellent for biomedical applications and incorporation into nanocomposites.^29^

Layered double hydroxides (LDHs), or anionic clays, are promising nanocarriers with a brucite-like structure and anion-exchange capability.^30,31^ Their structure of positively charged metal layers and interlayer anions provides high drug-loading, molecular protection, and stimuli-responsive release.^32–35^ Magnesium–aluminum LDH (Mg–Al LDH) is widely used in various therapies due to its biocompatibility and biodegradability in acidic environments.^36^

Perovskite oxides (ABO_3_) are important for drug delivery due to their unique crystal structure and multifunctional properties (*e.g.*, conductivity, magnetism).^37,38^ Comprised of larger A-site (*e.g.*, Ba^2+^, La^3+^) and smaller B-site (*e.g.*, Ti^4+^, Co^3+^) cations, they can form cubic, hexagonal, or rhombohedral structures and act as stimuli-responsive nanocarriers for controlled release.^39–41^

Despite advances in nanocarriers that improve drug bioavailability and controlled release, insufficient drug loading remains a major clinical challenge. To address this, three strategies exist: pre-loading (drug incorporated during synthesis), co-loading (drug integrated during formation), and post-loading, which is the focus of this study.^42–45^

In the post-loading strategy, nanocarriers with porous structures, high surface areas, and functionalizable sites are synthesized first, followed by drug loading.^46^ Through noncovalent interactions such as hydrophobic forces, hydrogen bonding, and π–π stacking, this method improves drug loading, stability, and reduces leakage. Compared with pre-loading and co-loading approaches, post-loading provides greater flexibility in drug selection, loading capacity, and process optimization.^47,48^

Taking advantage of the flexibility offered by the post-loading strategy, celecoxib was selected as the model therapeutic agent for this system. Celecoxib, a selective COX-2 inhibitor, is characterized by the following pharmacokinetic properties: oral bioavailability of 40–50%, peak plasma concentration achieved within 2–4 hours, plasma protein binding >97%, volume of distribution approximately 400 L, elimination half-life of 8–12 hours, and hepatic metabolism primarily *via* CYP2C9. These properties, together with its high lipophilicity (log *P* ≈ 3.5) and low aqueous solubility, render celecoxib an appropriate candidate for incorporation into a controlled-release pH-responsive nanocarrier system to enhance its therapeutic efficacy and minimize dose-related adverse effects.^49,50^

Therefore, the primary objectives of the present study are as follows: (i) to design and synthesize a novel LaFeO_3_/Mg–Al LDH/chitosan ternary nanocomposite *via* sol–gel, co-precipitation, and ultrasonication-assisted reflux methods; (ii) to comprehensively characterize the physicochemical, structural, morphological, and magnetic properties of the synthesized nanocomposite using FT-IR, XRD, SEM, EDS, elemental mapping, BET (pore size and surface area), and VSM techniques; (iii) to optimize the drug loading conditions namely pH, temperature, and water-to-ethanol volumetric ratio using Response Surface Methodology (RSM) coupled with Central Composite Design (CCD) in order to maximize both Drug Loading Efficiency (DLE) and Drug Loading Capacity (DLC); (iv) to evaluate the pH-responsive release behavior of celecoxib from the optimized nanocomposite in simulated physiological media (pH 5.8 and 7.4); (v) to investigate the swelling behavior of the nanocomposite as a function of pH; and (vi) to elucidate the underlying drug release kinetics by fitting the experimental data to zero-order, first-order, Higuchi, and Korsmeyer–Peppas kinetic models.

## 2. Experimental section

### 2.1. Chemicals and instrumentation

The starting materials required for sample preparation were procured from well-known suppliers, including Merck (Germany) and Fluka (Switzerland). To investigate the molecular structure and identify functional groups, Fourier-transform infrared (FT-IR) spectroscopy was performed in the frequency range of 400–4000 cm^−1^ using the potassium bromide (KBr) pellet method on a Magna 550 spectrometer (Nicolet). Detailed evaluation of surface properties, morphology, and elemental composition (including elemental mapping) was conducted using a field-emission scanning electron microscope (FE-SEM), model MIRA III, manufactured by TESCAN. To determine the crystalline structure and phase characteristics of the prepared materials, X-ray diffraction (XRD) patterns were recorded using an X'Pert Pro diffractometer (Panalytical, Netherlands) equipped with a Cu anode X-ray source (*λ* = 1.54 Å). The magnetic properties of the synthesized compounds were measured using a vibrating sample magnetometer (VSM), model T-9PPMS, produced by Magnetic Danesh-Pajouh Kashan. The pH of the environment was determined using a precision pH meter, model 827 pH (Metrohm, Switzerland). For efficient separation of sample components, an advanced centrifuge manufactured by Sakht Azma, capable of speeds up to 10 000 rpm, was employed. Furthermore, the optical absorption behavior of the materials was investigated using a UV-Vis spectrophotometer (UV-1800, Shimadzu, Japan), which measured light absorption and transmission in the UV and visible regions relative to a reference sample.

### 2.2. Preparation of precursors for drug carrier synthesis

#### 2.2.1. Synthesis of LaFeO_3_

For the targeted synthesis of perovskite LaFeO_3_, the sol–gel method was employed as a precise and controllable approach. Stoichiometric amounts of the metal precursors, namely lanthanum(iii) nitrate hexahydrate (La(NO_3_)_3_·6H_2_O) and iron(iii) nitrate nonahydrate (Fe(NO_3_)_3_·9H_2_O), were accurately weighed and dissolved in a specific volume of deionized water. The solvent volume was adjusted to achieve a final metal cation concentration of 0.1 M. After complete mixing and ensuring uniform dissolution of the salts, a stoichiometric amount of citric acid, serving as a complexing agent, was added to the solution at a 1 : 1 molar ratio with respect to the total metal ions under continuous stirring. The resulting solution was stirred at 75 °C to promote the formation of a foamy gel. The obtained gel was dried in an oven at 110 °C for 24 h to remove the solvent and stabilize the gel structure. The dried material was then ground and calcined in an electric furnace at 300 °C for 1 h to remove organic residues and volatile components. Finally, the resulting solid powder was calcined at 750 °C for 5 h to obtain the crystalline perovskite LaFeO_3_ phase.

#### 2.2.2. Synthesis of Mg–Al LDH

For the synthesis of Mg–Al LDH nanostructures, an aqueous solution containing magnesium nitrate (Mg(NO_3_)_2_·6H_2_O) and aluminum nitrate (Al(NO_3_)_3_·9H_2_O) in a molar ratio of 2 : 1 was prepared. To ensure complete mixing of the precursors, the solution was stirred using a high-speed magnetic stirrer for 6 h. For precise control of the precipitation conditions and the formation of the desired layered structure, a 1 M sodium hydroxide solution was added dropwise to the reaction mixture, and the pH was continuously maintained at 10 under constant stirring. Upon completion of the precipitation process, the solid phase was separated from the reaction medium by centrifugation at 6000 rpm for 10 min. To remove residual nitrate ions and other possible impurities, the solid precipitate was washed two times with deionized water. The resulting material was then dried in an oven at 90 °C for an adequate period, yielding pure Mg–Al LDH powders.

### 2.3. Preparation of drug carriers

#### 2.3.1. Synthesis of LaFeO_3_/chitosan nanocomposites

To synthesize LaFeO_3_/chitosan nanocomposites, 0.1 g of the LaFeO_3_ powder was dispersed in 10 mL of deionized water in a round-bottom flask at 25 °C for 20 minutes. Simultaneously, 0.1 g of finely ground chitosan was dissolved in 10 mL of a 2% (v/v) aqueous acetic acid solution and sonicated at 25 °C for 20 minutes to ensure complete dissolution. The chitosan solution was then gradually added to the LaFeO_3_ suspension, and the resulting mixture was stirred continuously under reflux at 80–85 °C for 2 hours. After completion of the reaction, the mixture was allowed to cool to room temperature, and the nanoparticles were separated by an external magnetic field. The collected nanoparticles were washed several times with deionized water to remove any residual impurities and were finally dried in an oven at 60 °C for 10 hours, yielding high-purity LaFeO_3_/chitosan nanocomposites.

#### 2.3.2. Preparation of chitosan/Mg–Al LDH nanocomposite

For the synthesis of chitosan/Mg–Al LDH nanocomposites, 1 g of the Mg–Al LDH was first completely dispersed in 10 mL of ethanol in a round-bottom flask at 25 °C for 20 minutes. Simultaneously, 1 g of thoroughly ground chitosan was dissolved in 10 mL of a 2% acetic acid aqueous solution and subjected to ultrasonic irradiation at 25 °C for 20 minutes to achieve complete dissolution. The chitosan solution was then gradually added to the Mg–Al LDH suspension, and the resulting mixture was continuously stirred continuously under reflux conditions at 80–85 °C for 2 hours. Upon completion of the process, the mixture was allowed to reach room temperature, and the nanocomposites were separated by centrifugation. The resulting nanoparticles were washed several times with deionized water to remove any possible impurities and, finally, the samples were dried in an oven at 60 °C for 10 hours to obtain high-purity chitosan/Mg–Al LDH nanocomposites.

#### 2.3.3. Preparation of LaFeO_3_/chitosan/Mg–Al LDH nanocomposite

To prepare the LaFeO_3_/chitosan/Mg–Al LDH nanocomposite, 0.1 g of the Mg–Al LDH was dispersed in 10 mL of ethanol. Simultaneously, 0.1 g of chitosan was dissolved in 10 mL of a 2% acetic acid solution, and 0.1 g of LaFeO_3_ was separately dispersed in 10 mL of deionized water. All solutions were subjected to ultrasonication for 20 minutes at 25 °C to achieve a uniform particle distribution. The solutions were then combined and stirred under reflux at 80 °C for 1 hour to allow the necessary interactions between the components to occur. After the mixture reached room temperature, the nanoparticles were separated by an external magnetic field, and the resulting samples were dried in an oven at 80 °C for 24 hours.

### 2.4. Drug loading procedure

#### 2.4.1. Construction of the calibration curve

**Fig. 1:**
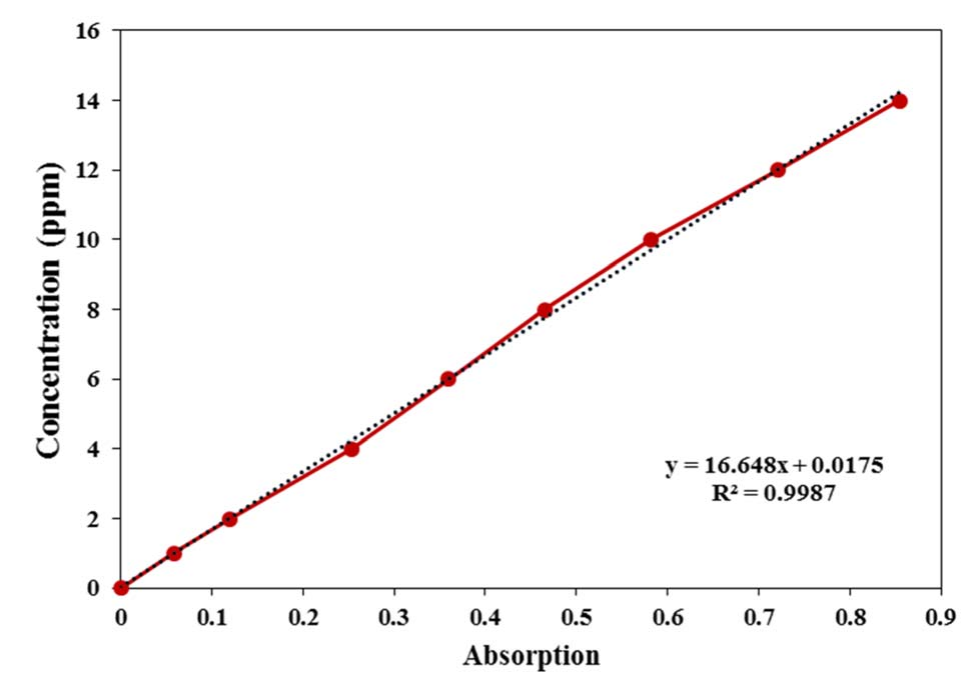
Calibration curve of celecoxib at 244 nm.

To quantitatively determine the concentration of celecoxib using UV-Vis spectrophotometry, the absorbance of samples was measured at the characteristic wavelength of 244 nm (the wavelength of maximum absorbance of celecoxib). For this purpose, a stock solution of celecoxib with a concentration of 100 ppm was initially prepared. To achieve this, 10 mg of celecoxib was dissolved in 10 mL of ethanol and then diluted to a final volume of 100 mL in a calibrated volumetric flask with a phosphate buffer (pH 7.4). From the stock solution, standard solutions with concentrations of 0, 1, 2, 4, 6, 8, 10, 12, and 14 ppm were prepared using a mixed solvent consisting of 10% (v/v) ethanol in phosphate buffer (pH 7.4) to cover the desired concentration range for calibration. The absorbance of each standard solution was measured at 244 nm using a spectrophotometer, and the data were used to construct the calibration curve (Fig. 1). The calibration curve was plotted based on the Beer–Lambert law (*i.e.*, the linear relationship between absorbance and concentration) and was employed to calculate the concentration of the drug released during the drug release experiments at specific time intervals.

#### 2.4.2. Preparation of celecoxib solutions and drug loading onto the carrier

To prepare the drug solutions, a stock solution of celecoxib with a concentration of 100 ppm was initially prepared. From this stock, diluted solutions with concentrations of 10, 20, 30, and 40 ppm were obtained according to the dilution equation (*C*_1_*V*_1_ = *C*_2_*V*_2_). For this purpose, 1, 2, 3, and 4 mL of the stock solution were transferred into separate volumetric flasks and diluted to a final volume of 10 Ml using a solvent mixture consisting of 10% (v/v) ethanol in phosphate buffer (pH 7.4). For the drug-loading experiments, 0.01 g of the carrier was dispersed in 10 mL of each celecoxib solution. The suspensions were maintained at room temperature under continuous stirring to promote drug adsorption. Samples were collected at predetermined time intervals (0, 6, 12, 24, and 36 h). Following centrifugation, the supernatant was separated, and the drug-loaded carriers were dried at room temperature. The residual concentration of celecoxib in the supernatant was determined using UV-Vis spectrophotometry at 244 nm. Based on the calibration curve, the amount of unabsorbed drug was calculated. Subsequently, the drug loading content and loading efficiency of the carrier were quantified using eqn (1) and (2).1
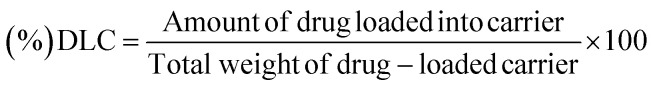
2
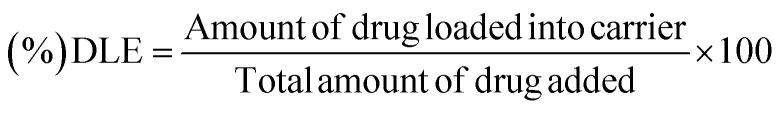


### 2.5. Optimization and modeling of drug loading using the RSM approach

To optimize and model the drug loading process onto the nanocarrier and its modeling, the Response Surface Methodology (RSM) was employed. The independent variables included process temperature, solution pH, and the volumetric ratio of water to ethanol, while Drug Loading Efficiency (DLE) and Drug Loading Capacity (DLC) were considered as the response variables. The experimental design was carried out using the Central Composite Design (CCD) in Design-Expert software (version 13.0.5.0). To determine the appropriate levels of the variables, a five-level factorial design with a range of *α* = ±2 was applied, the details of which are presented in Table 1. Based on these levels, the experimental matrix comprised 20 experiments with different combinations of operational conditions. To ensure the accuracy and reproducibility of the results, six replicate experiments were also included in the matrix, with full details provided in Table 2. The experiments were conducted under the designed laboratory conditions, and the DLE and DLC values were calculated and recorded precisely. The obtained data were then imported into Design-Expert software for data analysis and quantitative modeling of the process using the Response Surface Methodology. To elucidate the quantitative relationships between the independent variables and the responses (DLE and DLC), a second-order polynomial model was employed, expressed as follows:3
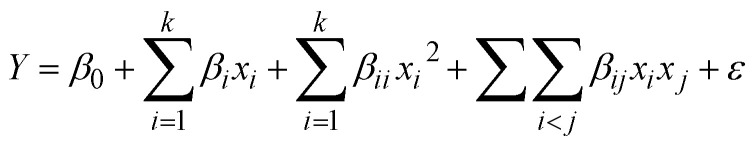
In this equation, *Y* represents the predicted response, *x*_*i*_ denotes the independent variables, and *x*_*i*_*x*_*j*_ indicates the first-order interaction effects between variables *x*_*i*_ and *x*_*j*_. The coefficients *β*_*i*_, *β*_*ii*_ and *β*_*ij*_ represent the linear, quadratic, and interaction effects of the variables, respectively, while *k* is the total number of factors under investigation, and *ε* denotes the experimental error. A comprehensive analysis of the results from the developed model was performed using an Analysis of Variance (ANOVA). This statistical method allowed for the evaluation of the significance of the modeling results and the determination of the influence of each variable and their interactions on the target responses.

**Table 1:** Levels of variables in coded and actual values for modeling the performance of the optimal nanocarrier

Independent variables		Level and range of variables				
		+*α*	+1	0	−1	−*α*
*A*	Temp. (°C)	50	43.9	35	26	20
*B*	pH	11	9.38	7	4.62	3
*C*	H_2_O/EtOH (v/v%)	95	76.75	50	23.25	5

**Table 2:** Experimental matrix designed for modeling the performance of the optimal nanocarrier using the CCD method

Run	pH	Temp. (°C)	H_2_O/EtOH (v/v%)
1	7	20	50
2	9.37841	26.0809	23.2428
3	9.37841	43.9191	76.7572
4	7	35	95
5	7	50	50
6	11	35	50
7	7	35	5
8	7	35	50
9	4.62159	26.0809	23.2428
10	7	35	50
11	7	35	50
12	7	35	50
13	9.37841	26.0809	76.7572
14	4.62159	43.9191	76.7572
15	3	35	50
16	4.62159	26.0809	76.7572
17	7	35	50
18	9.37841	43.9191	23.2428
19	7	35	50
20	4.62159	43.9191	23.2428

### 2.6. Swelling test

To quantitatively assess the swelling behavior of the optimized nanocarrier (the sample exhibiting the highest drug loading efficiency), a precise amount of the nanocarrier was individually weighed and immersed in glass containers containing 20 mL of buffer solutions with varying pH values (5.8 and 7.4). At one-hour intervals, the samples were removed from the aqueous medium, the surface water was removed using absorbent tissue, and they were accurately weighed before being returned to their container. The swelling ratio of the samples was calculated using eqn (4), where *W*_s_ represents the weight of the swollen sample and *W*_d_ denotes the weight of the dry sample.4
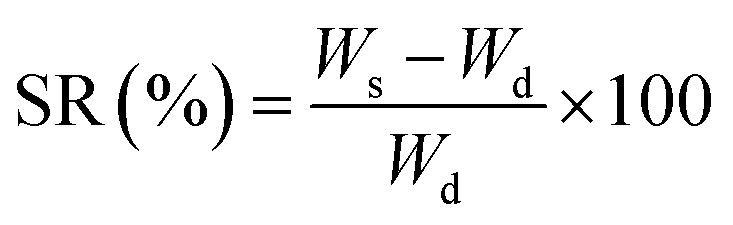


All experiments were performed in triplicate, and the results are presented as mean ± standard deviation (SD).

### 2.7. Drug release test

The release of the loaded drug from the optimized nanocarrier was investigated under laboratory conditions in environments with different pH values. To this end, phosphate buffers with pH values of 5.8 and 7.4 were employed. The drug release study was performed at pH 7.4 to simulate normal physiological conditions (*e.g.*, blood circulation) and at pH 5.8 to simulate the slightly acidic microenvironment typical of inflamed tissues. This comparative approach was utilized to evaluate the pH-responsive delivery capabilities of the nanocomposite.

The release study was conducted using a conventional static immersion method, which is widely accepted in the literature for initial screening of nanocarrier-based drug delivery systems. Although the study was not performed in strict compliance with pharmacopoeial standards (*e.g.*, USP dissolution apparatus I or II), the method employed is consistent with standard protocols reported for similar nanocarrier systems.

For the drug release assessment, 10 mg of the drug-loaded optimized nanocarrier was dispersed in 50 mL of the buffer solution in a sealed glass container maintained at 37 ± 0.5 °C under continuous stirring at 100 rpm. At predetermined time intervals (1, 2, 4, 6, 8, 12, and 24 hours), 5 mL of the solution was sampled. After centrifugation at 6000 rpm for 10 minutes to separate the nanocarrier particles, the absorbance of the supernatant was measured using a UV-Vis spectrophotometer (UV-1800, Shimadzu, Japan) at *λ*_max_ = 244 nm. After each sampling, an equivalent volume (5 mL) of fresh pre-warmed buffer was added to the dissolution medium to maintain a constant total volume (sink condition). The concentration of the released drug was determined based on the linear equation derived from the calibration curve, and these data were used to calculate the percentage of celecoxib drug release over time. All experiments were performed in triplicate, and the results are presented as mean ± standard deviation (SD).

## 3. Results and discussion

### 3.1. Catalyst characterization

**Fig. 2:**
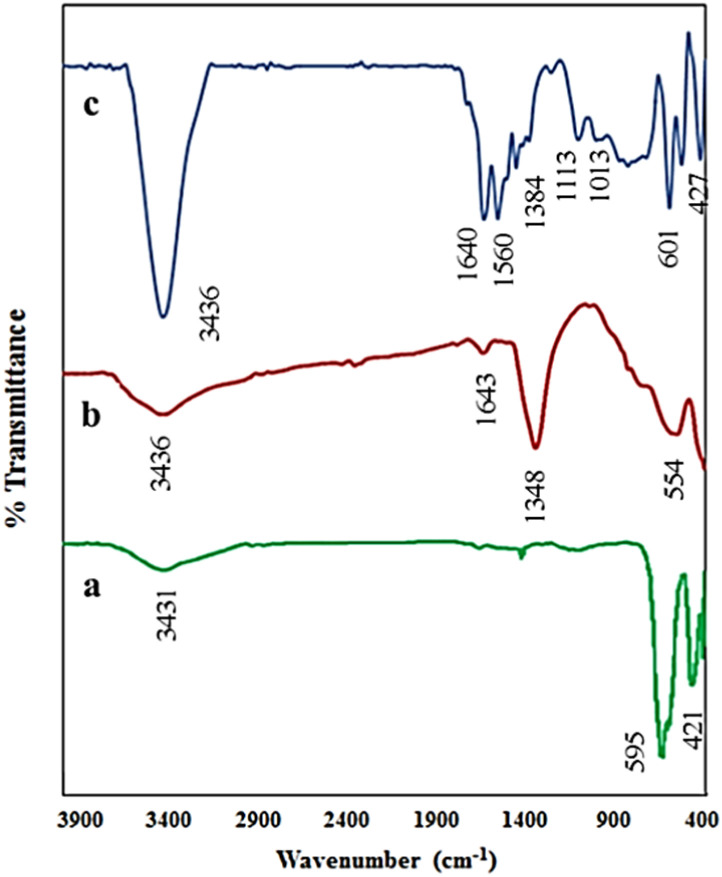
FT-IR spectra of (a) LaFeO_3_, (b) Mg–Al LDH and (c) LaFeO_3_/chitosan/Mg–Al LDH composite.

The FT-IR spectra of the synthesized samples were analyzed based on their characteristic absorption bands to accurately evaluate the structures and chemical compositions of the materials (Fig. 2). For the LaFeO_3_ sample (Fig. 2a), the broad absorption band observed in the range of 3400–3450 cm^−1^ is assigned to the stretching vibrations of hydroxyl (O–H) groups, mainly arising from surface-adsorbed moisture or the presence of hydroxyl groups on the particle surfaces. The most prominent spectral features of this sample are the well-defined bands in the region of 400–600 cm^−1^, which correspond to the stretching vibrations of Fe–O and La–O bonds in the perovskite octahedral framework, confirming the successful formation of the perovskite phase. In the spectrum of Mg–Al LDH (Fig. 2b), the broad bands located in the region of 3400–3500 cm^−1^ are attributed to the stretching vibrations of layer hydroxyl groups and interlayer water molecules. The band observed around 1630 cm^−1^ is associated with the bending vibrations of interlayer water. Moreover, the distinct bands in the range of 1350–1380 cm^−1^ indicate the stretching vibrations of nitrate (NO_3_^−^) groups, which act as the dominant interlayer anions in the LDH structure. In addition, the bands below 800 cm^−1^ correspond to the metal–oxygen vibrations (Mg–O and Al–O), further confirming the layered structure of the compound. The FT-IR spectrum of the LaFeO_3_/chitosan/Mg–Al LDH composite (Fig. 2c) displays a combination of the spectral features of all three components. The broad absorption band in the region of 3400–3500 cm^−1^ originates from the overlapping stretching vibrations of hydroxyl groups (from LDH and chitosan) and amino groups of chitosan. The band in the region of 1630–1650 cm^−1^, in addition to adsorbed water, is attributed to the vibrations of chitosan amine groups. The absorption band in the range of 1350–1380 cm^−1^ further confirms the presence of interlayer nitrate ions. Moreover, the bands observed in the region of 1000–1150 cm^−1^ are assigned to the stretching vibrations of C–O and C–N bonds of chitosan. Finally, the persistence of the characteristic absorption bands in the range of 500–700 cm^−1^ demonstrates the stability of the LaFeO_3_ perovskite and Mg–Al LDH phases within the composite structure. Collectively, these results confirm the successful formation of the ternary composite and the retention of the structural features of each component in the final product.

**Fig. 3:**
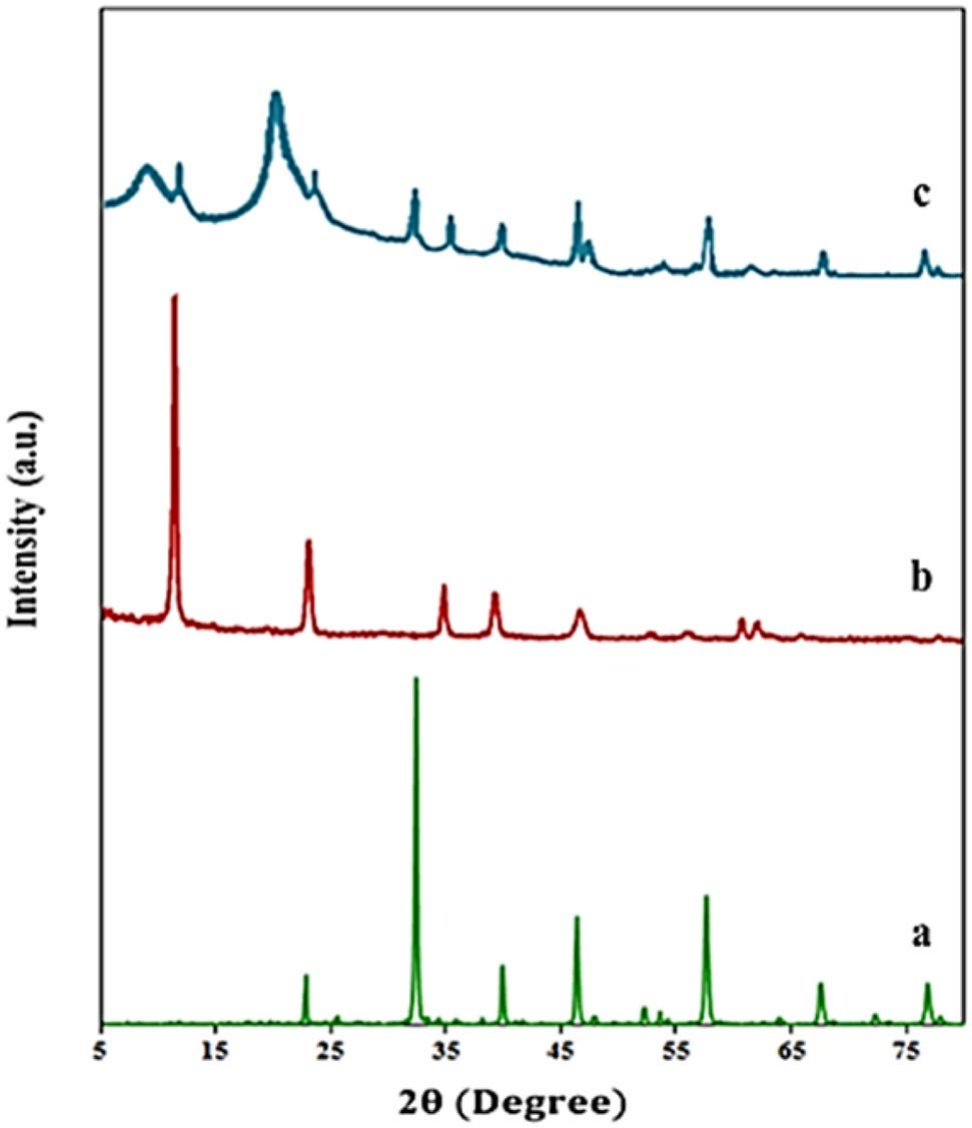
XRD patterns of LaFeO_3_ (a), Mg–Al LDH (b) and LaFeO_3_/chitosan/Mg–Al LDH nanocomposite (c).

Fig. 3 presents the X-ray diffraction (XRD) patterns of the synthesized samples. As shown, pattern (a) corresponds to LaFeO_3_, pattern (b) corresponds to Mg–Al LDH, and pattern (c) corresponds to the LaFeO_3_/chitosan/Mg–Al LDH nanocomposite. In pattern (a), the characteristic peaks appear at 2*θ* values of 22.9°, 32.3°, 40.0°, 46.2°, 57.8°, 67.4°, and 77.0°, which correspond to the (101), (121), (202), (220), (242), (044), and (204) crystal planes, respectively. These peaks are in full agreement with the standard LaFeO_3_ pattern (JCPDS No. 37-1493), indicating the successful formation of a pure perovskite structure in the synthesized sample. In pattern (b), sharp and well-defined peaks are observed at 2*θ* values of 11.3°, 23.2°, 35.1°, 39.6°, 47.1°, 60.8°, and 62.4°, corresponding to the (003), (006), (012), (015), (018), (110) and (113) planes, respectively. These peaks match the standard Mg–Al LDH pattern (JCPDS No. 38-0487) and confirm the presence of a well-ordered layered structure with defined interlayer spacing characteristic of hydrotalcite. In pattern (c), in addition to the characteristic peaks of the LaFeO_3_ and Mg–Al LDH phases, two additional peaks appear at around 10° and 20°, corresponding to the amorphous and semi-crystalline structures of chitosan. These peaks confirm the presence of chitosan in the nanocomposite. Furthermore, the reduction in peak intensity and the broadening observed in this pattern compared to that of the pure samples may be attributed to interactions between the components and the presence of the a chitosan polymeric network. Overall, the XRD results presented in Fig. 3 clearly confirm the successful and simultaneous formation of LaFeO_3_, Mg–Al LDH, and chitosan phases in the final structure, and the complete agreement of the peaks with standard reference data verifies the accuracy of the synthesized structures.

**Fig. 4:**
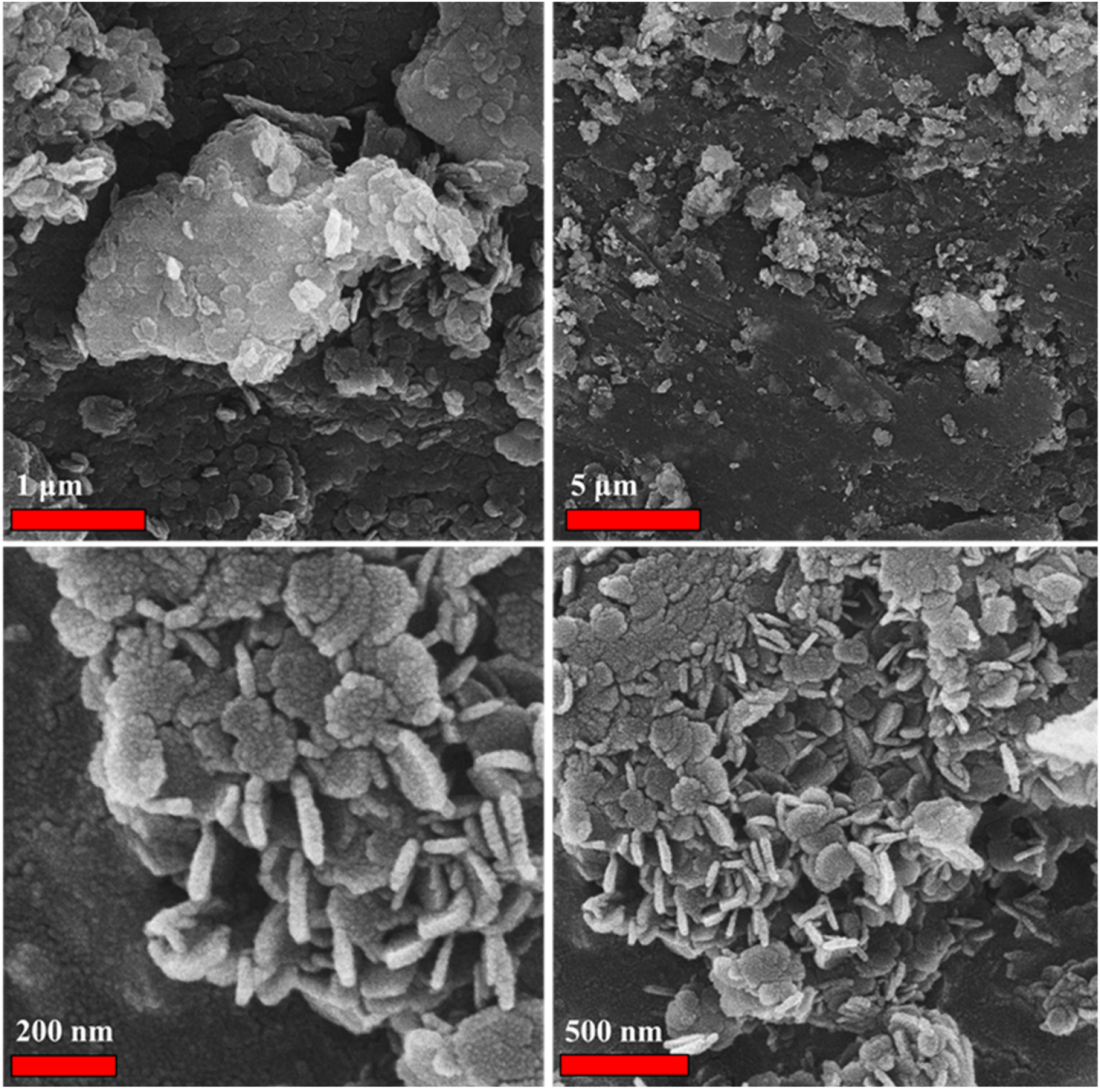
SEM images of the LaFeO_3_/chitosan/Mg–Al LDH nanocomposite.

The scanning electron microscopy (SEM) image of the LaFeO_3_/chitosan/Mg–Al LDH ternary nanocomposite is presented in Fig. 4. As observed in the SEM image, the morphology of the synthesized sample predominantly consists of nanosheets and plate-like structures with a relatively ordered and aggregated arrangement. This sheet-like structure clearly indicates the presence of the Mg–Al LDH phase, which, in accordance with the literature, appears as thin, layered sheets. In addition to these plates, particles with relatively irregular or quasi-spherical shapes are also observed, likely originating from the LaFeO_3_ phase or from the chitosan polymer matrix. The uniform dispersion of these phases throughout the matrix demonstrates the a successful synthesis process and the effective interaction between the components of the nanocomposite. Moreover, the absence of severe aggregation of the plates and their homogeneous distribution confirm the role of chitosan as a dispersing and stabilizing agent. Overall, the morphology observed in the SEM image is consistent with the expected structure of the LaFeO_3_/chitosan/Mg–Al LDH ternary nanocomposite, indicating the formation of a sheet–particle architecture with a high surface area and well-dispersed components, which can contribute to the enhanced functional properties of the nanocomposite.

**Fig. 5:**
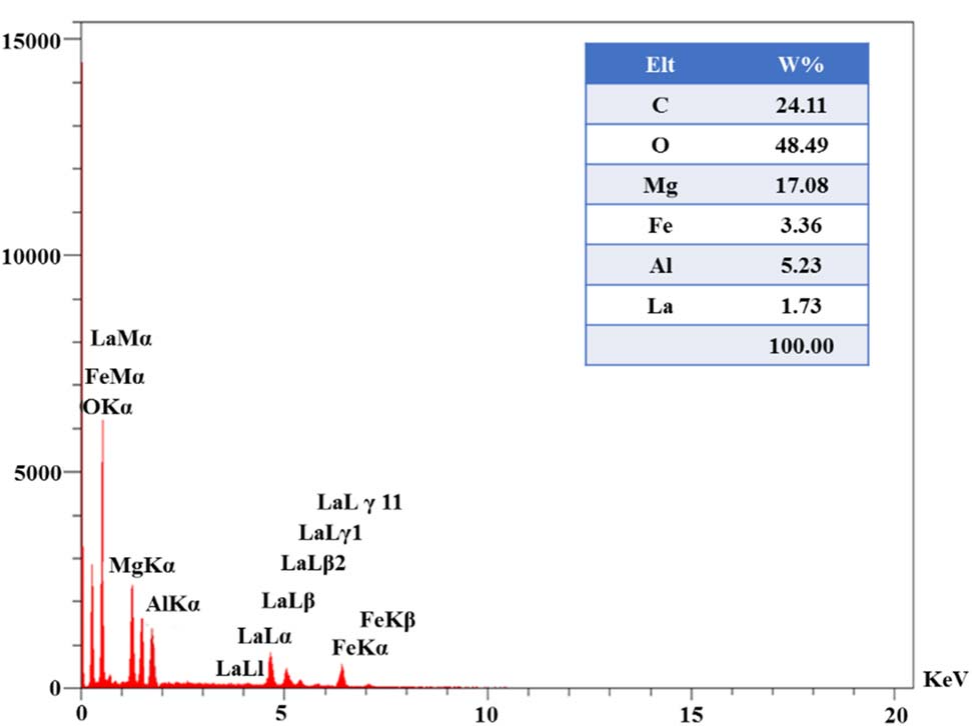
EDX analysis of the LaFeO_3_/chitosan/Mg–Al LDH nanocomposite.

The EDS analysis of the LaFeO_3_/chitosan/Mg–Al LDH composite is presented in Fig. 5. As observed in the spectrum, the primary constituent elements of the composite include carbon, oxygen, magnesium, aluminum, nitrogen, iron, and lanthanum, each representing distinct components of the composite structure. Oxygen exhibits the highest intensity in the spectrum, which is attributed to the presence of hydroxyl groups, carbonate ions, and the oxide structures of the composite's constituents. Carbon, a major component of chitosan and the carbonate ions within the LDH layers, is also prominent in the spectrum. Magnesium and aluminum indicate the presence of the Mg–Al LDH phase, while lanthanum and iron clearly confirm the formation of the LaFeO_3_ phase within the composite structure. These results validate the successful and homogeneous integration of the various components into the studied composite sample.

**Fig. 6:**
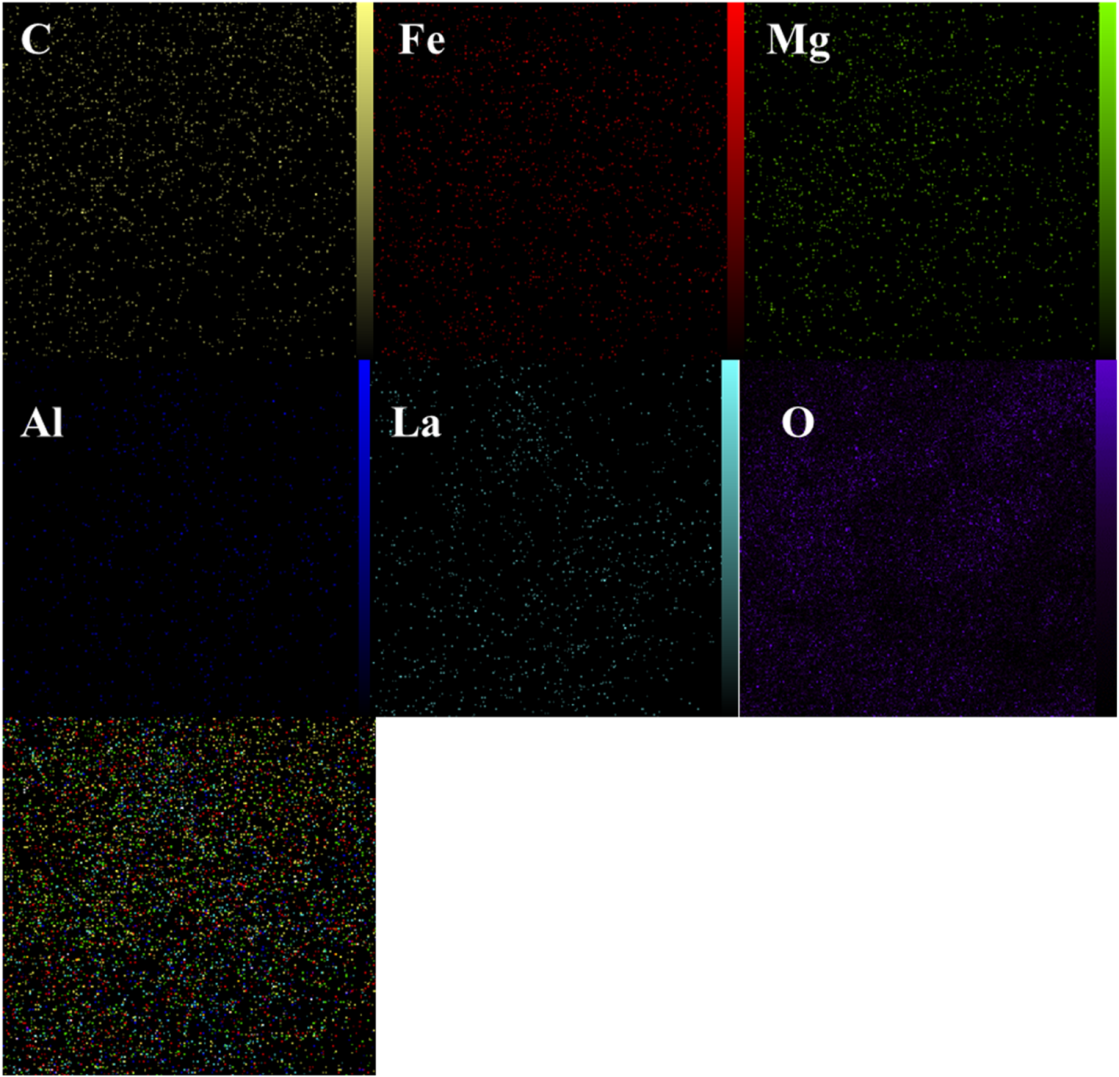
Elemental mapping analysis of the LaFeO_3_/chitosan/Mg–Al LDH nanocomposite.

Fig. 6 shows the elemental mapping of the LaFeO_3_/chitosan/Mg–Al LDH nanocomposite. The maps of C, O, La, Mg, Al, and Fe are presented individually, as well as in a combined map. As observed, all elements are uniformly and homogeneously distributed across the sample surface. This uniform distribution indicates successful synthesis and effective integration of the organic and inorganic components within the final composite structure. The absence of aggregation or phase separation further confirms the high quality of the synthesis and the favorable distribution of the phases, which contributes to the enhanced functional properties and stability of the nanocomposite.

**Fig. 7:**
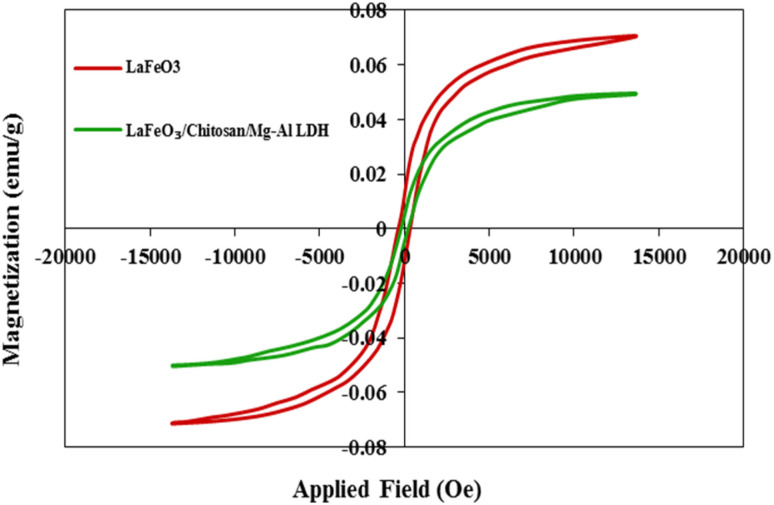
Magnetization-field curves of LaFeO_3_ samples and LaFeO_3_/chitosan/Mg–Al LDH nanocomposite.

In Fig. 7, the magnetization curves of the LaFeO_3_ sample and the LaFeO_3_/chitosan/Mg–Al LDH nanocomposite are presented as a function of the applied magnetic field (using VSM). As observed, the LaFeO_3_ sample exhibits pronounced ferromagnetic behavior, characterized by higher saturation magnetization and a more distinct hysteresis loop compared to those of the nanocomposite. This behavior is consistent with the perovskite structure, which generates significant magnetization due to the Fe–O–Fe superexchange interactions. In contrast, the LaFeO_3_/chitosan/Mg–Al LDH nanocomposite shows a significant reduction in saturation magnetization and a narrower hysteresis loop. This reduction is primarily attributed to the dilution of the magnetic LaFeO_3_ phase by the addition of non-magnetic chitosan and Mg–Al LDH components, leading to decreased magnetic interactions and, consequently, to a lower overall magnetization of the sample.

**Fig. 8:**
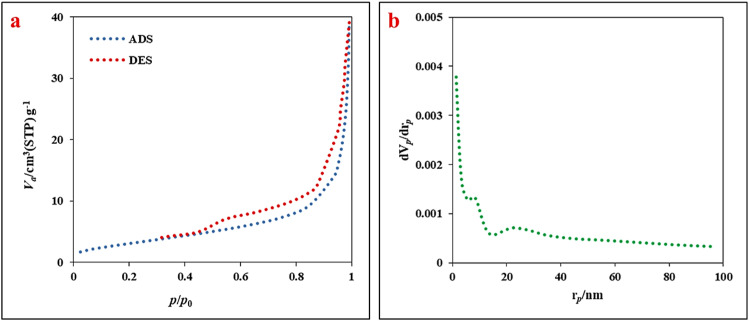
(a) N_2_ adsorption–desorption isotherms and (b) BJH pore size distribution of the LaFeO_3_/chitosan/Mg–Al LDH nanocomposite.

The Brunauer–Emmett–Teller (BET) surface area and Barrett–Joyner–Halenda (BJH) pore size distribution of the optimized LaFeO_3_/chitosan/Mg–Al LDH ternary nanocomposite were determined *via* nitrogen adsorption–desorption isotherms. The adsorption–desorption isotherms and pore size distribution curves are presented in Fig. 8a and b, respectively. The nanocomposite exhibited a specific surface area of 11.391 m^2^ g^−1^, a total pore volume of 0.0593 cm^3^ g^−1^ (at *P*/*P*_0_ = 0.990), and a mean pore diameter of 21 nm. The pore size distribution falls within the mesoporous range (2–50 nm), which is particularly suitable for celecoxib molecules (molecular dimensions of approximately 1–2 nm), thereby facilitating efficient drug loading and subsequent controlled release. These structural characteristics, combined with the optimized loading conditions (pH = 9.38, temperature = 43.92 °C, water : ethanol ratio = 76.76), contribute to the exceptionally high drug loading efficiency of 98.72% and loading capacity of 43.96%.

**Fig. 9:**
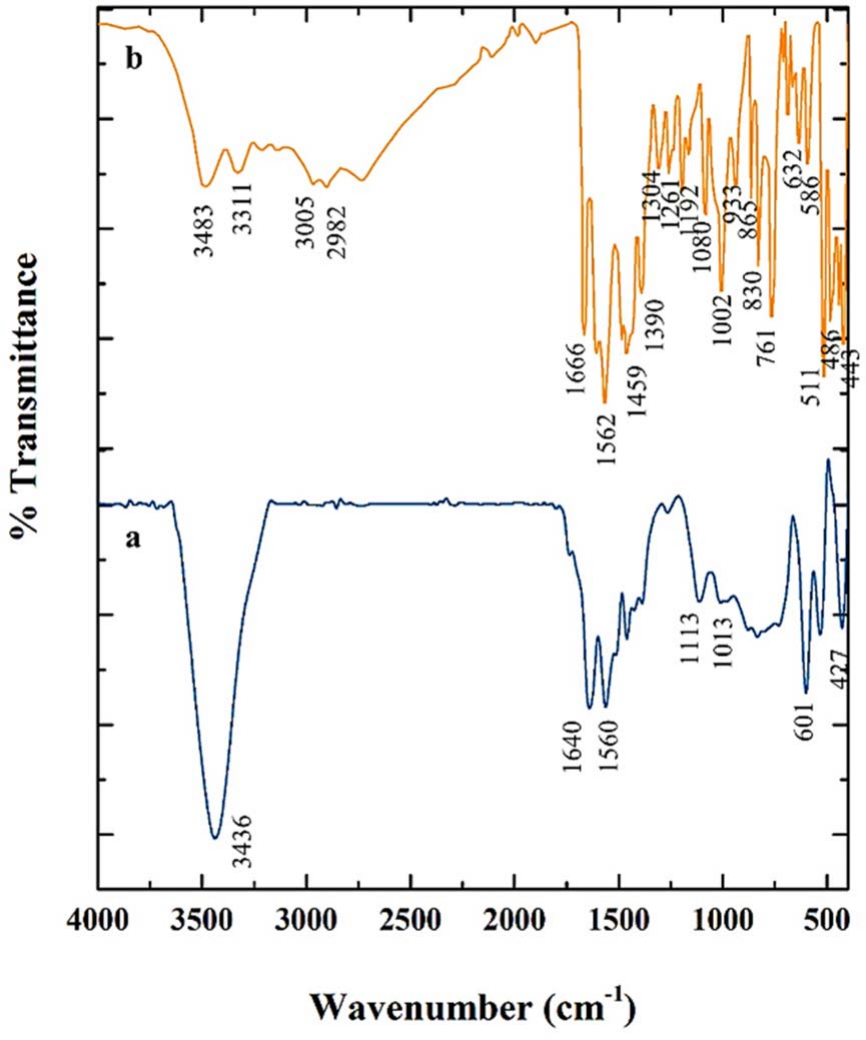
FTIR spectra of (a) the LaFeO_3_/chitosan/Mg–Al LDH nanocarrier and (b) the drug-loaded nanocarrier.

To investigate the chemical interactions and the successful loading of the drug into the nanocarrier, FT-IR analysis was performed. Fig. 9 shows the FT-IR spectra of the LaFeO_3_/chitosan/Mg–Al LDH nanocomposite (spectrum a) and the drug-loaded nanocomposite (spectrum b). In the spectrum of the pure carrier (Fig. 9a), characteristic bands are observed at 3400–3500 cm^−1^ (O–H and N–H stretching), 1630–1650 cm^−1^ (N–H bending), 1350–1380 cm^−1^ (NO_3_^−^), 1000–1150 cm^−1^ (C–O/C–N stretching), and 500–700 cm^−1^ (metal–oxygen bonds). After drug loading (Fig. 9b), the spectrum clearly exhibits new absorption bands associated with the functional groups of the drug. The peaks appearing at 3483 and 3311 cm^−1^ are attributed to the NH_2_ stretching vibrations. The bands at 3005 and 2982 cm^−1^ correspond to the C–H stretching vibrations of sp^2^ and sp^3^ hybridized carbons, respectively. In addition, the characteristic aromatic C=C stretching peaks are observed at 1666 and 1562 cm^−1^, while the bands at 1390 and 1080 cm^−1^ are assigned to the S=O functional groups of the drug. The appearance of these distinct peaks, along with the shifting of the intrinsic bands of the carrier, provides strong evidence for the successful loading of the drug into the nanocarrier matrix.

### 3.2. Evaluation of celecoxib loading capacity and adsorption trends onto nanocarriers

**Fig. 10:**
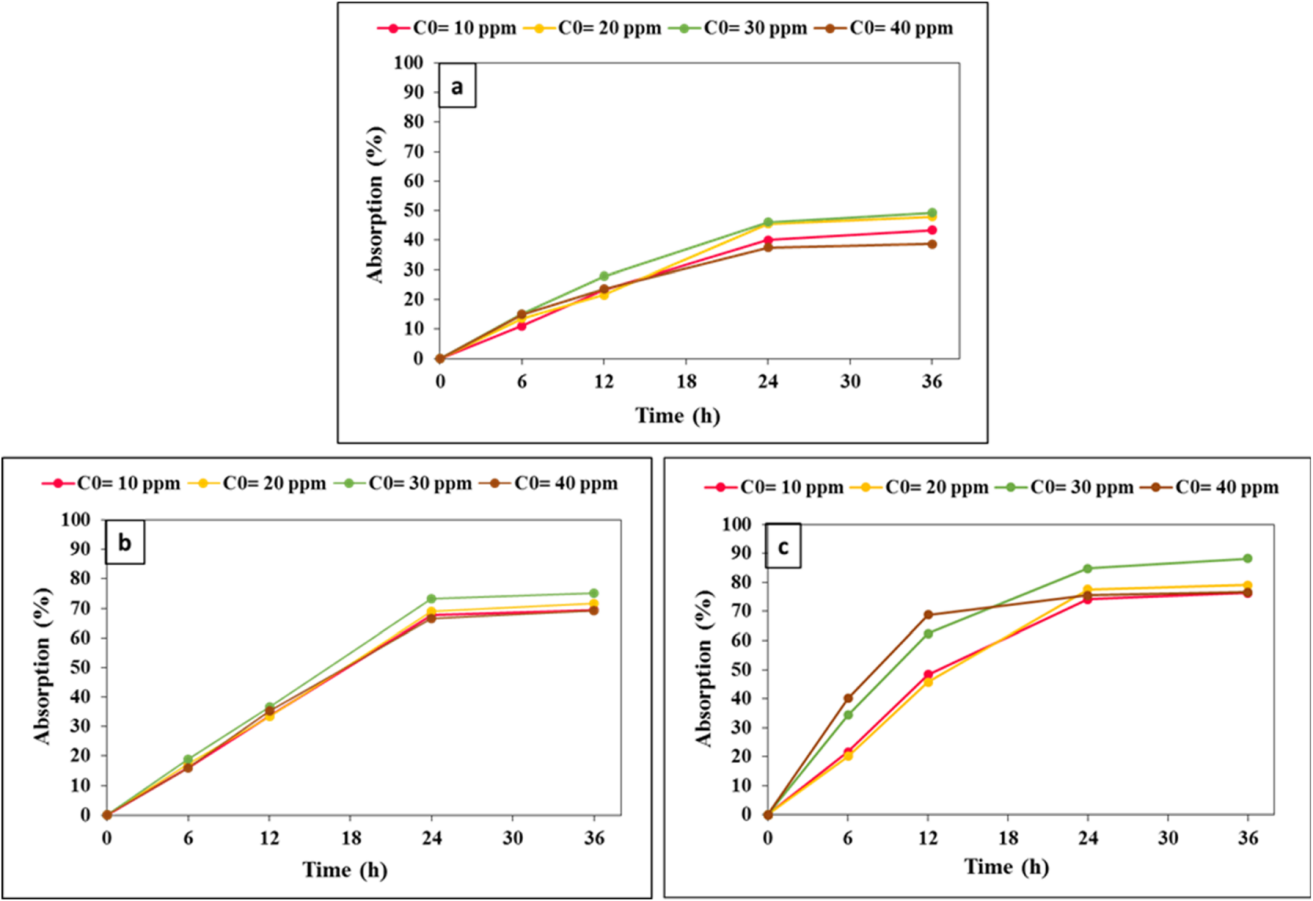
Comparison of celecoxib adsorption percentage trends at different time intervals and initial drug concentrations for three nanocarriers: LaFeO_3_/chitosan (a), chitosan/Mg–Al LDH (b) and LaFeO_3_/chitosan/Mg–Al LDH (c).

The loading of celecoxib onto three nanocarriers, namely chitosan/Mg–Al LDH, LaFeO_3_/chitosan, and LaFeO_3_/chitosan/Mg–Al LDH, was investigated at initial concentrations of 10, 20, 30, and 40 ppm over time intervals of 0, 6, 12, 24, and 36 hours. Fig. 10 illustrates the changes in the residual celecoxib concentration in the solution following the loading process for each nanocarrier. Across all nanocarriers, the drug adsorption increased steadily up to 24 hours, after which it reached relative equilibrium between 24 and 36 hours, indicating saturation of the nanocarriers' loading capacity. As the initial drug concentration increased from 10 to 30 ppm, the adsorption amount rose significantly rose; however, at 40 ppm, the changes in adsorption were negligible.

**Fig. 11:**
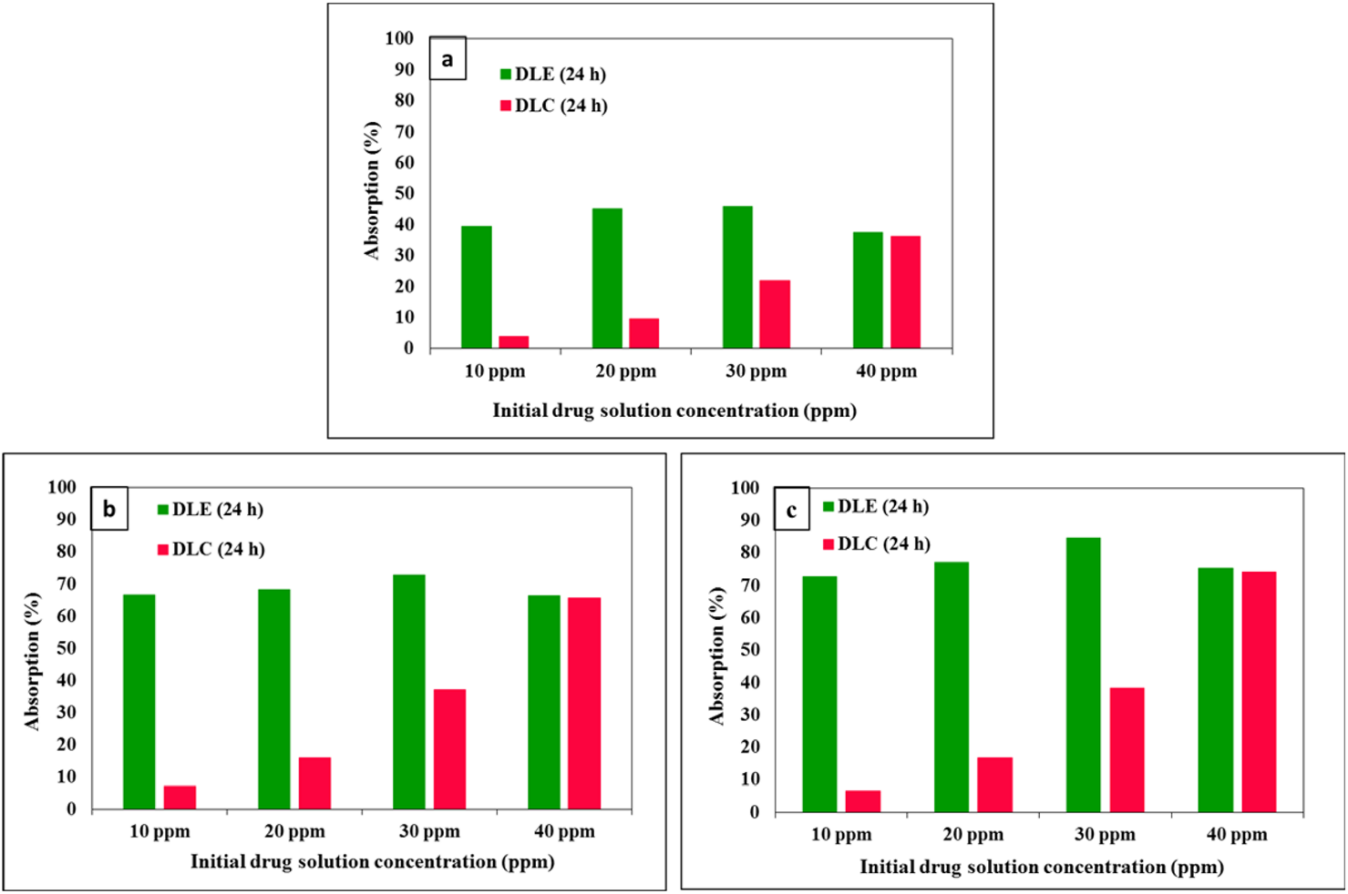
Comparison of drug loading efficiency and drug loading content variations for 0.01 g of three nanocarriers: LaFeO_3_/chitosan (a), chitosan/Mg–Al LDH (b) and LaFeO_3_/chitosan/Mg–Al LDH (c).

Fig. 11 presents the drug loading efficiency (DLE%) and drug loading content (DLC%) for 0.01 g of each nanocarrier at various concentrations. The DLE% peaked at an initial concentration of 30 ppm but exhibited a slight decrease at 40 ppm. In contrast, the DLC% consistently increased with higher initial drug concentrations, reflecting an increase in the amount of drug loaded relative to the nanocarrier weight.

Comparative analysis of the nanocarriers revealed that the ternary nanocomposite LaFeO_3_/chitosan/Mg–Al LDH exhibited the highest drug adsorption, DLE%, and DLC% across all tested concentrations. Specifically, at the optimal concentration of 30 ppm, this nanocarrier demonstrated the highest loading efficiency.

### 3.3. Experiment design and performance modeling of the LaFeO_3_/chitosan/Mg–Al LDH nanocomposite

To enhance the adsorption efficiency of celecoxib onto the LaFeO_3_/chitosan/Mg–Al LDH nanocomposite, the effects of operational variables, including solution pH (3–11), temperature (20–50 °C), and water : ethanol volumetric ratio (5–95%), were investigated using Response Surface Methodology (RSM). Previous studies reported an adsorption efficiency of 88.19% for this adsorbent, suggesting potential for further improvement through optimization. A Central Composite Design (CCD) with *α* = ±2 was employed to design a matrix of 20 experiments (Table 3). Drug Loading Efficiency (DLE) and Drug Loading Capacity (DLC) were defined as the response variables.

**Table 3:** Results of the experimental matrix designed using the central composite design (CCD) method

Run	pH	Temp.	H_2_O/EtOH (v/v%)	DLE (24 h)	DLC (24 h)
1	7	20	50	77.3	35.1
2	9.37841	26.0809	23.2428	68.8	30
3	9.37841	43.9191	76.7572	96.8	43.8
4	7	35	95	93.4	42.3
5	7	50	50	91.1	41.2
6	11	35	50	88.6	40.1
7	7	35	5	58.4	28
8	7	35	50	84.6	38.3
9	4.62159	26.0809	23.2428	62.3	28.2
10	7	35	50	85.9	39.2
11	7	35	50	82.5	34.8
12	7	35	50	86.2	39
13	9.37841	26.0809	76.7572	88.5	42
14	4.62159	43.9191	76.7572	81.4	36.9
15	3	35	50	65.7	29.9
16	4.62159	26.0809	76.7572	77.8	35.5
17	7	35	50	85.9	38.9
18	9.37841	43.9191	23.2428	73.9	35
19	7	35	50	86.1	39.2
20	4.62159	43.9191	23.2428	62.5	29

The relationship between the independent variables and the responses was modeled using second-order polynomial equations derived using the least squares regression method (eqn (5) and (6)). In these equations, temperature, pH, and the water : ethanol ratio were denoted as *A*, *B*, and *C*, respectively.

5 DLE = +85.27 + 6.04*A* + 2.96*B* + 9.95*C* + 1.20*AB* + 1.02*AC* + 0.8250*BC* − 3.31*A*^2^ − 0.8204*B*^2^ − 3.75*C*^2^

6 DLC = +38.26 + 2.81*A* + 1.41*B* + 4.40*C* + 0.5750*AB* + 0.7000*AC* − 0.3250*BC* − 1.43*A*^2^ − 0.2237*B*^2^ − 1.82*C*^2^

### 3.4. Analysis of variance

**Fig. 12:**
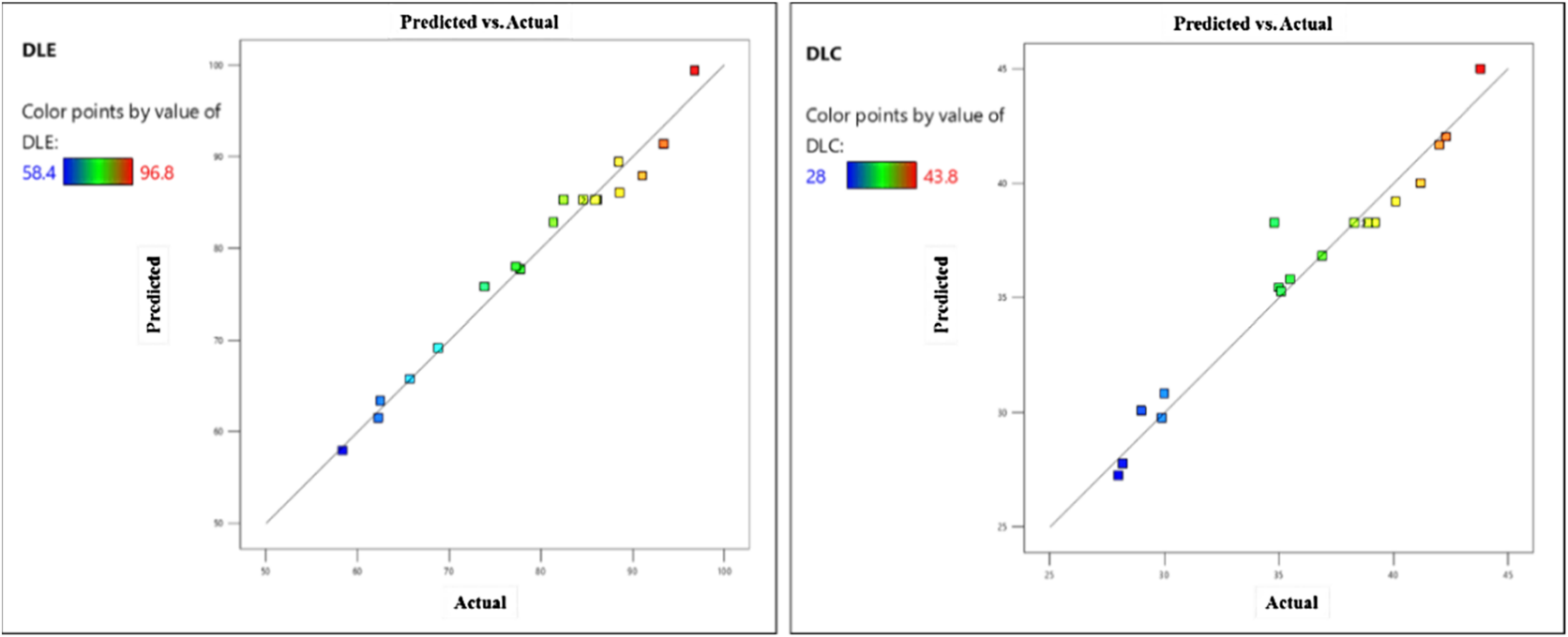
Comparison of actual and predicted values of the model for the responses DLE and DLC.

Analysis of variance (ANOVA) was conducted to evaluate the statistical significance of the models (Tables 4 and 5). The results indicated high model significance (*p*-value < 0.0001) with determination coefficients (*R*^2^) exceeding 0.9, confirming excellent agreement between the experimental and predicted data. The Lack of Fit was non-significant (*p* > 0.05), and the Adequate Precision values above 4 further validated the models' reliability. The ANOVA results revealed that pH and the water : ethanol ratio had the most significant effects on DLE and DLC, as indicated by their high *F*-values. Fig. 12 illustrates the close agreement between actual and predicted values for both responses. Optimal conditions were identified at pH 7, 35 °C, and a 50% water : ethanol ratio, yielding maximum DLE and DLC. These findings demonstrate that the developed models are robust tools for predicting and optimizing the adsorption process.

Analysis of variance (ANOVA) results for DLE on the LaFeO_3_/chitosan/Mg–Al LDH carrier

**Table:** 

Source	Sum of squares	Df	Mean square	*F*-Value	*p*-Value	
Model	2324.56	9	258.28	55.66	<0.0001	Significant
*A*-pH	498.53	1	498.53	107.44	<0.0001	
*B*-TEMP	119.56	1	119.56	25.77	0.0005	
*C*-v% ETOH	1351.61	1	1351.61	291.28	<0.0001	
*AB*	11.52	1	11.52	2.48	0.1462	
*AC*	8.40	1	8.40	1.81	0.2081	
*BC*	5.44	1	5.44	1.17	0.3041	
*A* ^2^	158.17	1	158.17	34.09	0.0002	
*B* ^2^	9.70	1	9.70	2.09	0.1788	
*C* ^2^	203.19	1	203.19	43.79	<0.0001	
Residual	46.40	10	4.64			
Lack of fit	35.96	5	7.19	3.44	0.1004	Not significant
Pure error	10.44	5	2.09			
Cor total	2370.97	19

**Table:** 

Model statistics summary			
Std. dev.	2.15	*R* ^2^	0.9804
Mean	79.89	Adjusted *R*^2^	0.9628
*C* V%	2.70	Predicted *R*^2^	0.8788
PRESS	N/A	Adeq precision	27.2207

Analysis of variance (ANOVA) results for DLC on the LaFeO_3_/chitosan/Mg–Al LDH carrier

**Table:** 

Source	Sum of squares	Df	Mean square	*F*-Value	*p*-Value	
Model	451.41	9	50.16	23.37	<0.0001	Significant
*A*-pH	107.72	1	107.72	50.18	<0.0001	
*B*-TEMP	27.16	1	27.16	12.65	0.0052	
*C*-v% ETOH	264.04	1	264.04	123.01	<0.0001	
*AB*	2.64	1	2.64	1.23	0.2930	
*AC*	3.92	1	3.92	1.83	0.2064	
*BC*	0.8450	1	0.8450	0.3937	0.5444	
*A* ^2^	25.78	1	25.78	12.01	0.0061	
*B* ^2^	0.7214	1	0.7214	0.3361	0.5749	
*C* ^2^	23.77	1	23.77	11.08	0.0076	
Residual	21.47	10	2.15			
Lack of fit	6.77	5	1.35	0.4609	0.7923	Not significant
Pure error	14.69	5	2.94			
Cor total	472.87	19

**Table:** 

Model statistics summary			
Std. dev.	1.47	*R* ^2^	0.9546
Mean	36.32	Adjusted *R*^2^	0.9138
*C* V%	4.03	Predicted *R*^2^	0.8408
PRESS	N/A	Adeq precision	17.1313

### 3.5. Response surface analysis

In this study, two-dimensional (2D) and three-dimensional (3D) plots for each pair of key variables in the celecoxib drug loading process using 0.01 g of the LaFeO_3_/chitosan/Mg–Al LDH nanocomposite are presented based on DLE and DLC within a unified framework.

#### 3.5.1. Effects on drug loading efficiency (DLE)

Initially, 2D contour and 3D response surface methodology (RSM) plots derived from the experimental design were used to comprehensively analyze variations in drug loading efficiency (DLE), considering three critical variables: temperature, pH, and the volumetric water : ethanol ratio. Each plot reflects the system's behavior under simultaneous changes in two variables while keeping the third variable fixed at its central value. Owing to the nature of RSM modeling, these plots enable both quantitative and qualitative assessment of the individual and interactive effects of the variables.

**Fig. 13:**
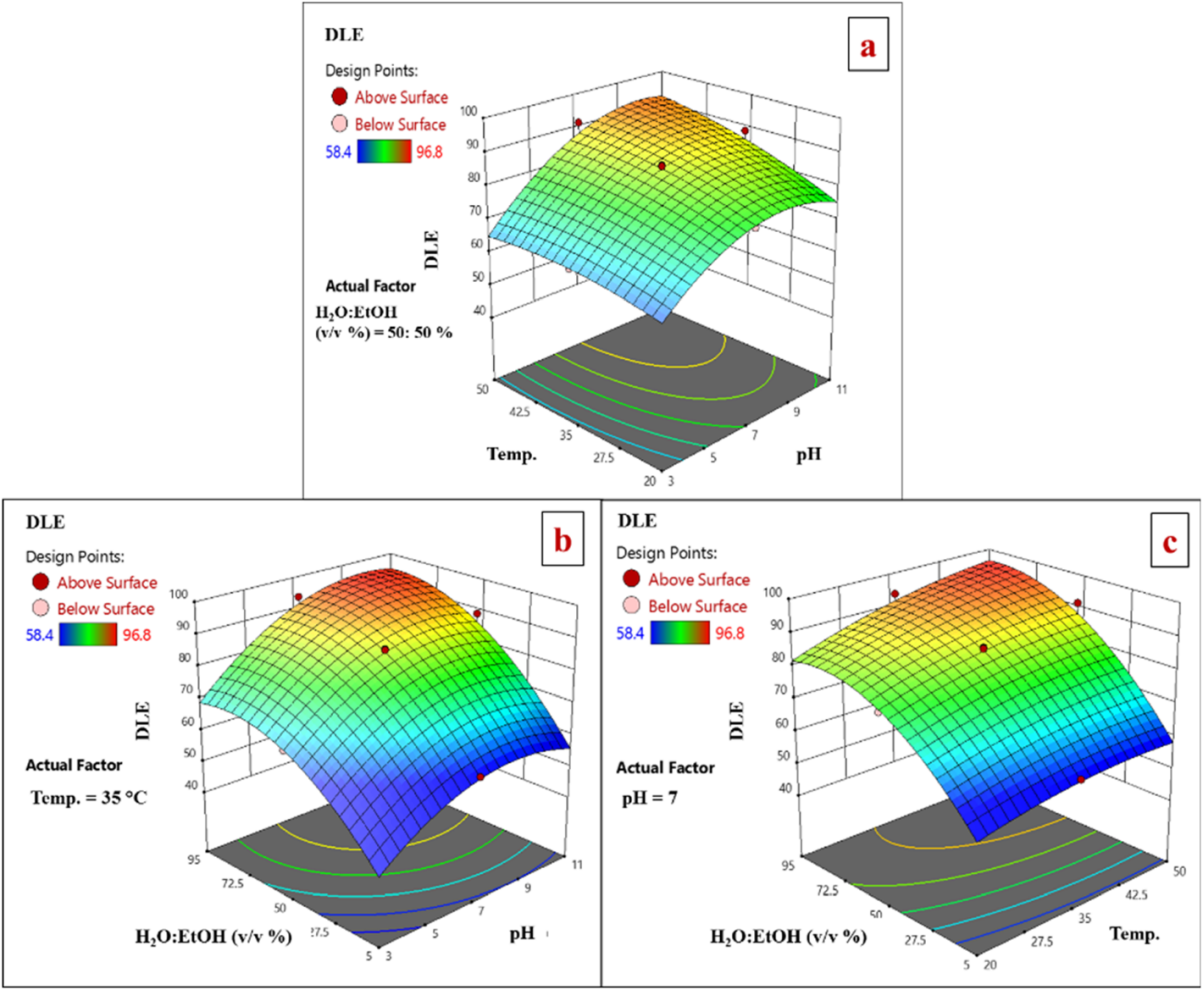
2D and 3D response surface analysis plots for DLE using LaFeO_3_/chitosan/Mg–Al LDH nanocomposite.

The combined influence of temperature and pH on DLE, at a fixed water : ethanol ratio of 50 : 50, reveals an upward trend in DLE toward regions of elevated temperature and pH. This enhancement can be attributed to the heightened kinetic energy of drug molecules at higher temperatures, which promotes diffusion into the adsorbent's pores, coupled with pH-induced alterations in the surface charges of both the adsorbent and drug, fostering favorable electrostatic interactions in alkaline environments. The predominantly concentric and uniform contour lines indicate a relatively weak interaction between temperature and pH, with each variable exerting an independent, additive positive effect on DLE (Fig. 13a). Subsequently, the interplay between pH and the volumetric water : ethanol percentage on DLE, under constant temperature (35 °C), demonstrates improvements with increasing pH and water fraction. This pattern arises from enhanced solvent polarity at higher water content, which boosts drug solubility and the mass transfer to the adsorbent surface, alongside pH-driven charge modifications that strengthen electrostatic attractions. The concentric contour patterns underscore a weak interaction, confirming the independent and augmentative roles of these variables (Fig. 13b). Furthermore, the concurrent effects of temperature and the water : ethanol ratio on DLE, at a fixed pH of 7, exhibit progressive increases with both variables. This is linked to amplified molecular mobility of the drug at elevated temperatures and improved solvent polarity with greater water proportions, accelerating adsorption. The regular, concentric contours reflect minimal interaction, highlighting the standalone contributions of each factor to DLE elevation (Fig. 13c). Overall, these plots illustrate the predominance of individual variable effects on DLE, with no pronounced strong interactions, as evidenced by consistent contour configurations. Such insights emphasize the critical role of precise control over temperature, pH, and solvent composition in optimizing the adsorption process.

#### 3.5.2. Effects on drug loading content (DLC)

**Fig. 14:**
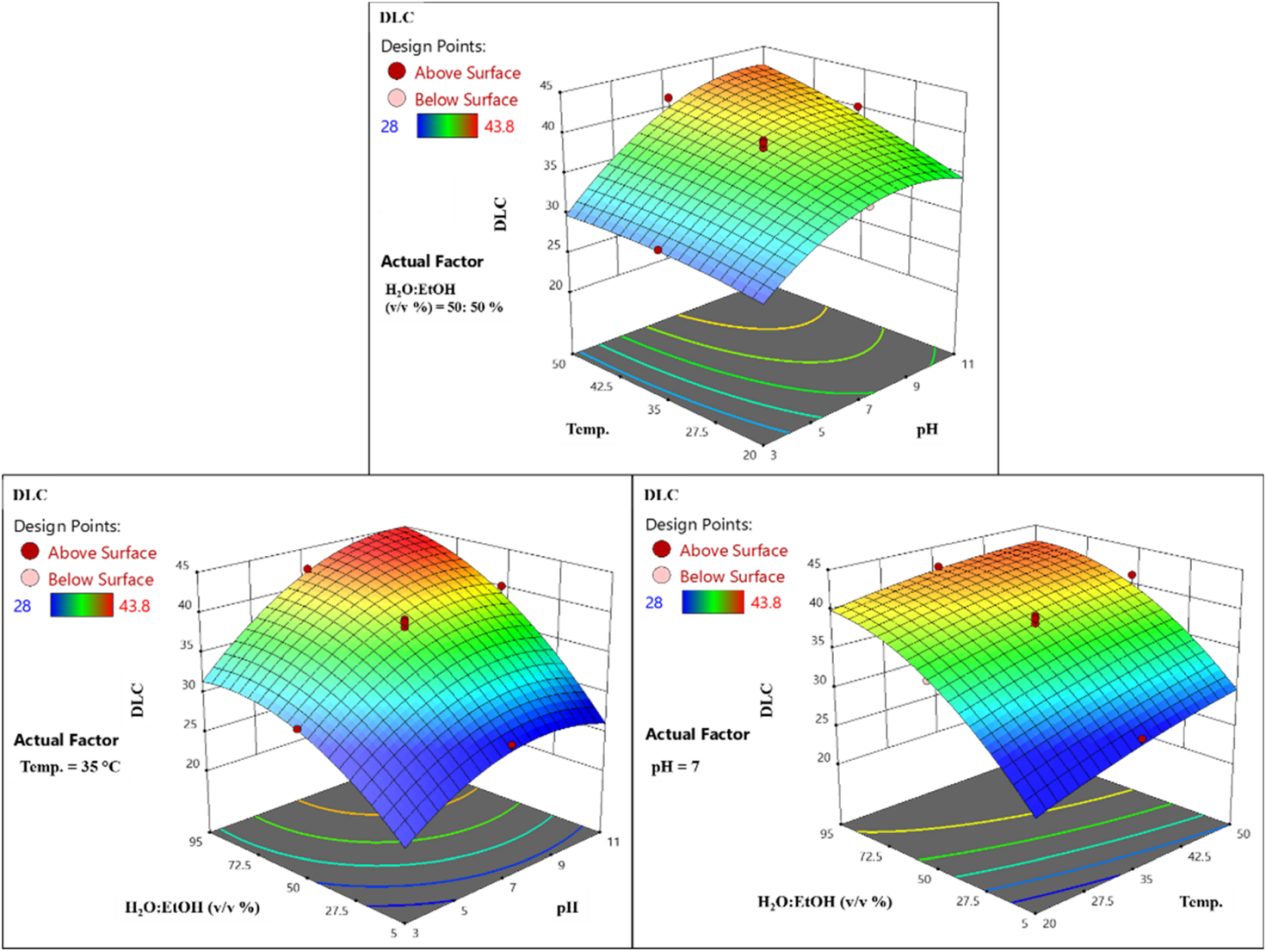
2D and 3D response surface analysis plots for DLC using LaFeO_3_/chitosan/Mg–Al LDH nanocomposite.

The simultaneous impact of temperature and pH on DLC, with the water : ethanol fixed at 50%, shows a steady ascending trajectory, underscoring the positive influences of both factors. This improvement stems from reinforced drug–adsorbent interactions and increased molecular mobility at higher temperatures, as well as the greater structural stability of the adsorbent in more alkaline conditions (Fig. 14a). In addition, the combined effects of pH and the water : ethanol ratio on DLC, at constant temperature, indicate continuous growth with rising levels of both, contrasting with reductions at low pH or high ethanol content. This behavior points to a positive interactive effect, where alkaline media and elevated water fractions enhance effective interactions through optimized charge dynamics and solvent polarity (Fig. 14b). Finally, the combined effect of temperature and the water : ethanol ratio on drug loading content (DLC) at a fixed pH of 7 is analyzed (Fig. 14c). Simultaneous increases in temperature and water fraction result in a consistent rise in DLC, reflecting their positive impact on loading efficiency. Higher temperatures enhance drug loading, while increased water content improves DLC, unlike higher ethanol levels, which reduce the efficiency. These findings indicate a synergistic interaction between temperature and solvent composition, significantly boosting DLC.

### 3.6. Pareto analysis

**Fig. 15:**
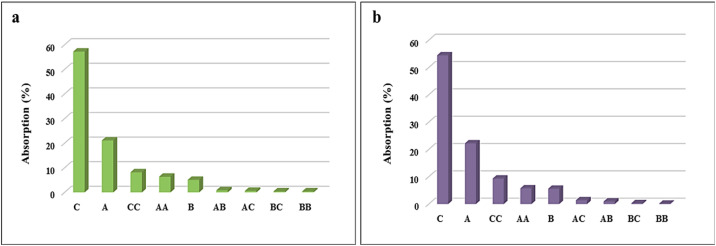
Pareto analysis results of DLE (a) and DLC (b).

Pareto analysis was employed as a quantitative method to determine the contribution of each independent factor and its interactive effects on the system response. Based on this analysis, the percentage impact of each factor was calculated using the regression model coefficients and the mathematical relationship provided in eqn (7):7
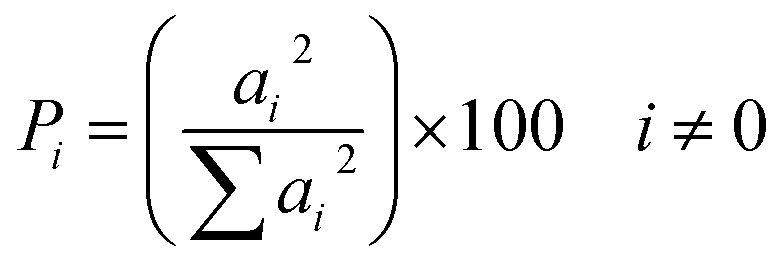
where *P*_*i*_ represents the percentage impact of each factor, and *a*_*i*_ is the coefficient associated with that factor in the optimized model. This analysis was applied to the surface adsorption process of celecoxib drug on 0.01 g of the LaFeO_3_/chitosan/Mg–Al LDH nanocomposite in both DLE and DLC modes, with results presented in Fig. 15.

According to Fig. 15a for drug loading efficiency (DLE), variable *C* (volume percentage of water–ethanol) is the primary factor with a 57% contribution, as the water-to-ethanol ratio affects drug solubility, surface interactions, and thermodynamic equilibrium. Variable *A* (temperature) ranks second with 21%, indicating the process's temperature dependence. Other factors such as *B* (pH) and their interactions have lesser impacts. In Fig. 15b for drug loading content (DLC), variable *C* dominates with 54%, influencing chemical interactions through solvent composition. Variable *A* plays a key role with 22%, while pH and other interactions show limited effects (below 10%), attributable to the stability of the nanocomposite in the pH range. Overall, solvent composition (*C*) and temperature (*A*) are the key factors, with nonlinear interactions like CC highlighting the need for precise optimization. These results confirm the critical role of nanoscale structure in the dynamics of surface adsorption.

### 3.7. Perturbation curve

**Fig. 16:**
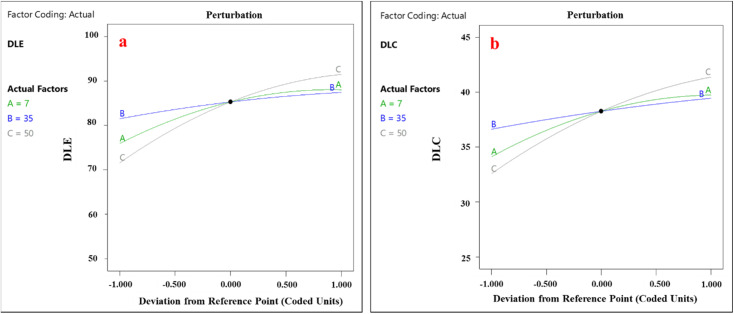
Perturbation plot obtained for (a) DLE and (b) DLC.

The perturbation plot is an analytical tool that enables the evaluation of the impact of each factor at a specific point, particularly the central point of the design space. Using this plot, the manner and extent of each variable's influence can be examined individually. To construct the perturbation plot, the system response is calculated under conditions where only one variable changes while others remain fixed. This approach allows for a precise analysis of the model's sensitivity to variations in each factor. The perturbation plots for drug loading efficiency and drug loading content are presented in Fig. 16, clearly illustrating the influence of each variable at the central point. The results indicate that all components have a significant impact on the performance of the LaFeO_3_/chitosan/Mg–Al LDH nanocomposite in celecoxib loading, but variables *C* (volume percentage of water : ethanol) and *B* (pH) exhibit the greatest contributions, identifying them as key factors. These findings align with the temperature-dependent nature of loading processes and the importance of solvent composition for thermodynamic equilibrium.

### 3.8. Optimization of process parameters for celecoxib adsorption

To identify the optimal conditions for the adsorption of celecoxib onto the LaFeO_3_/chitosan/Mg–Al LDH composite, which outperformed other adsorbents in this study, a design of experiments (DoE) framework was implemented using the Design Expert software. The RSM was applied to simultaneously optimize multiple process parameters, targeting maximum DLE and DLC. The investigated variables included solution pH, temperature, and the ethanol-to-water volume ratio.

The optimized conditions and corresponding predicted responses are summarized in Table 6. The model indicated that a pH of 9.38, a temperature of 43.92 °C, and an ethanol-to-water ratio of 76.76 yielded optimal performance, with predicted DLE and DLC values of 99.38% and 44.98%, respectively, achieving a desirability score of 1.000. To confirm the reliability of these predictions, experiments were conducted under the proposed optimal conditions. The experimental outcomes, with a DLE of 98.72% and a DLC of 43.96%, closely matched the model predictions, demonstrating the robustness and precision of the RSM-based model in optimizing the adsorption process.

**Table 6:** Effect of process variables on LaFeO_3_/chitosan/Mg–Al LDH performance modeling

Process conditions			Model predictions			Experimental results	
pH	Temperature (°C)	Ethanol : water ratio	DLE	DLC	Desirability	DLE	DLC
9.378	43.919	76.757	99.382	44.983	1.000	98.72	43.96

### 3.9. Drug release evaluation

The release behavior of celecoxib from the optimized LaFeO_3_/chitosan/Mg–Al LDH nanocomposite, selected for its superior drug loading efficiency, was investigated in phosphate buffer solutions at pH 5.8 and 7.4. The drug release was monitored over 24 hours using UV-Vis spectroscopy to quantify the released drug concentration, with calculations based on a pre-calibrated standard curve.

**Fig. 17:**
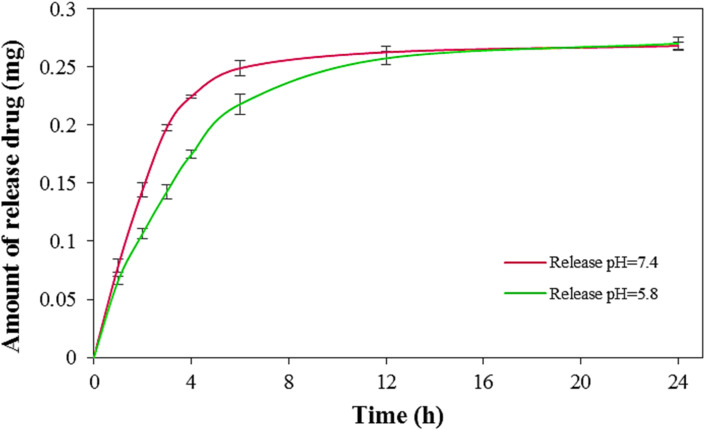
Release of celecoxib from the LaFeO_3_/chitosan/Mg–Al LDH nanocomposite (mean ± SD, *n* = 3).

As illustrated in Fig. 17, the release kinetics exhibited a biphasic pattern in both pH environments. The initial phase, spanning the first 4–8 hours, was characterized by a rapid release, likely due to the desorption of surface-bound or loosely associated celecoxib molecules. This burst release can be attributed to the protonation of chitosan's amino groups, which facilitates ion exchange and enhances drug release in both acidic and neutral conditions. Subsequently, a slower, sustained release phase was observed, approaching a plateau, indicative of the gradual liberation of interlayer drug molecules with stronger interactions with the nanocomposite. Comparative analysis revealed that the initial release rate was higher at pH 7.4, resulting in a greater cumulative release within the first 8 hours compared to that at pH 5.8. This observation, while seemingly contradictory to the higher swelling ratio at pH 5.8, can be explained by the predominance of electrostatic repulsion between the negatively charged drug and the nanocomposite surface at pH 7.4, as opposed to electrostatic attraction at pH 5.8 (see Section 3.12 for detailed mechanistic discussion). However, after 12 hours, the total drug release in both environments converged to approximately 90% of the loaded drug, indicating that long-term release is governed primarily by diffusion rather than by surface charge effects. The pH-responsive nature of the LaFeO_3_/chitosan/Mg–Al LDH nanocomposite was evident, with enhanced release rates in the acidic environment (pH 5.8), aligning with conditions typical of tumor microenvironments. This pH sensitivity, coupled with the nanocomposite's structural stability and strong drug-carrier interactions, minimizes premature drug release in neutral physiological conditions (pH 7.4), promoting preferential release under acidic conditions. These characteristics position the nanocomposite as a promising platform for stimuli-responsive drug delivery, offering controlled and sustained release tailored to specific physiological environments.

### 3.10. Swelling behavior of the nanocomposite

The swelling behavior of the optimized LaFeO_3_/chitosan/Mg–Al LDH nanocomposite, selected for its exceptional drug loading efficiency, was evaluated to assess its water absorption capacity and structural responsiveness in aqueous environments. Swelling tests were conducted in phosphate buffer solutions at pH 5.8 and pH 7.4. The swelling ratio (SR), calculated using eqn (4), quantifies the extent of water uptake by the nanocomposite over time.

**Fig. 18:**
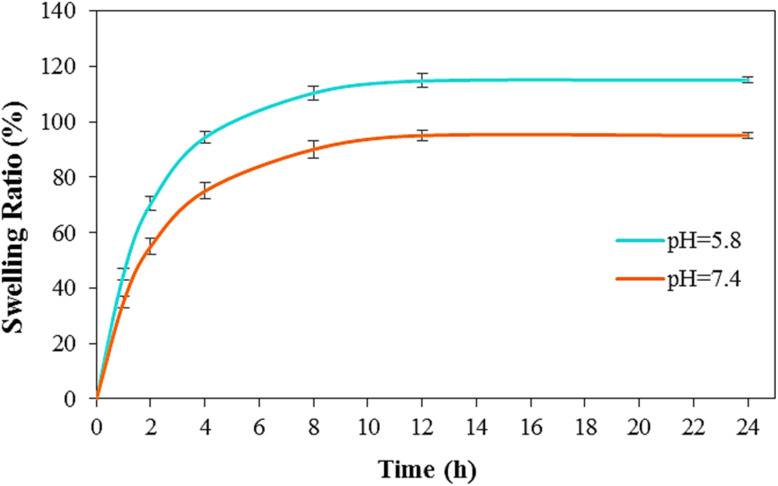
Time-dependent swelling behavior of LaFeO_3_/chitosan/Mg–Al LDH nanocomposite in phosphate buffer solutions at varying pH levels (mean ± SD, *n* = 3).

As shown in Fig. 18, the swelling ratio increased rapidly in both pH environments during the initial phase, reaching equilibrium after approximately 12 hours. At pH 5.8, the nanocomposite exhibited a higher swelling ratio compared to that at pH 7.4, likely due to the protonation of chitosan's amino groups, which enhances electrostatic repulsion and promotes water absorption into the polymeric network. In contrast, at pH 7.4, the swelling ratio was lower, indicating greater structural stability and controlled water uptake, attributed to the reduced ionization of functional groups in the neutral environment. This pH-dependent swelling behavior arises from the synergistic ionic and hydrogen-bonding interactions among the chitosan matrix, Mg–Al LDH layers, and LaFeO_3_ phase, which facilitate network expansion and water retention.

The observed swelling characteristics highlight the nanocomposite's pH-responsive behavior, with enhanced water uptake under acidic conditions, indicating its potential for pH-triggered drug release. The ability to achieve rapid initial swelling followed by equilibrium, together with its pH-dependent swelling behavior, highlights the potential of the LaFeO_3_/chitosan/Mg–Al LDH nanocomposite as a pH-responsive controlled drug delivery platform.

### 3.11. Kinetics of celecoxib release from LaFeO_3_/chitosan/Mg–Al LDH nanocomposite

**Fig. 19:**
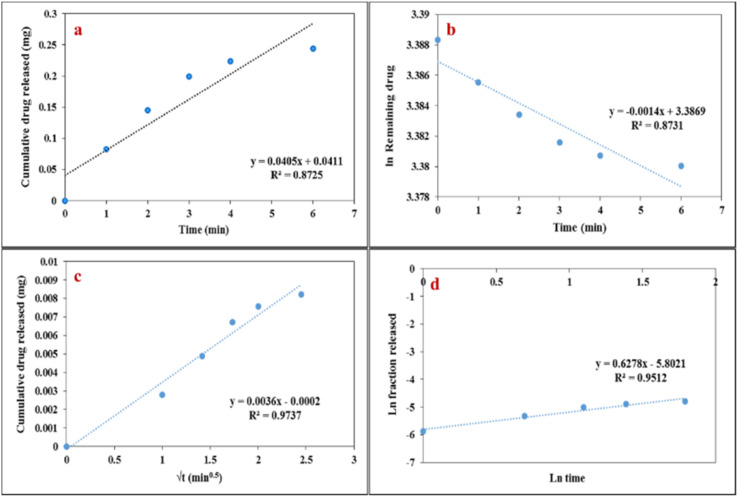
Evaluation of celecoxib release kinetics from LaFeO_3_/chitosan/Mg–Al LDH nanocomposite based on (a) zero-order, (b) first-order, (c) Higuchi and (d) Korsmeyer–Peppas models.

In this study, the release kinetics of celecoxib from the LaFeO_3_/chitosan/Mg–Al LDH nanocomposite at pH 7.4 were investigated using four common kinetic models, including the zero-order, first-order, Higuchi, and Korsmeyer–Peppas models (Fig. 19). Data analysis was performed over a time range of 0–6 hours, as the system approaches equilibrium beyond this period, and no significant changes in the release rate are observed. The kinetic analysis revealed that the Higuchi model provided the highest correlation coefficient (*R*^2^ = 0.9737), suggesting that molecular diffusion is the dominant driving force for celecoxib release from the nanocomposite matrix into the surrounding medium. On the other hand, the Korsmeyer–Peppas model yielded a release exponent value of *n* = 0.62 (*R*^2^ = 0.9512). Since 0.45 < *n* < 0.89, this indicates an anomalous (non-Fickian) transport mechanism. Rather than a contradiction, this combination suggests a coupled mechanism: while diffusion is the primary mode of drug transport (explaining the excellent Higuchi fit), the concurrent swelling and structural relaxation of the chitosan/LDH nanocomposite matrix also plays a significant role. Therefore, the overall release is governed by both diffusion and polymer relaxation. This complex behavior can be well attributed to the network-like structure, hybrid composition, and electrostatic interactions between the drug and the carrier. In contrast, the zero-order and first-order models demonstrated weaker correlations, with *R*^2^ values of 0.8725 and 0.8731, respectively. These findings indicate that drug release from the studied nanocomposite does not follow simple linear or exponential patterns but is instead governed by more complex mechanisms involving controlled release and structural constraints. Table 7 presents the kinetic constants (*k*) and determination coefficients (*R*^2^) for the different models describing the release behavior of celecoxib from the LaFeO_3_/chitosan/Mg–Al LDH nanocomposite.

**Table 7:** Results of the kinetic modeling of celecoxib release from the LaFeO_3_/chitosan/Mg–Al LDH nanocomposite

Kinetic model	Key parameters	*R* ^2^
Zero-order	*k*_0_ = 0.0405 mg h^−1^	0.8725
First-order	*k*_1_ = 0.0014 h^−1^	0.8731
Higuchi	*k*_H_ = 0.0036 mg h^−0.5^	0.9737
Korsmeyer–Peppas	*k* = 0.003021, *n* = 0.62	0.9512

### 3.12. Correlation between swelling and drug release behavior

The reviewer raised an important point regarding the apparent discrepancy between the swelling profiles (Fig. 18) and drug release profiles (Fig. 17). While the swelling ratio was higher at pH 5.8 compared to pH 7.4, the initial drug release rate was faster at pH 7.4. This seemingly contradictory observation requires careful mechanistic explanation.

#### 3.12.1. Mechanism 1: electrostatic interactions dominate over swelling

At pH 7.4, the chitosan amino groups (p*K*_a_ ≈ 6.3–6.5) remain largely deprotonated, while the nanocomposite surface carries negative charges derived from LaFeO_3_ hydroxyl groups and LDH surface anions. Celecoxib, with a p*K*_a_ of approximately 11.1, exists predominantly in its ionized (negatively charged) form at pH 7.4. The resulting electrostatic repulsion between the negatively charged drug and the negatively charged nanocomposite surface promotes rapid drug desorption and release during the initial hours. Conversely, at pH 5.8, chitosan amino groups become protonated (NH_3_^+^), imparting a net positive charge to the nanocomposite surface. This positive surface charge creates electrostatic attraction with the partially negative or neutral celecoxib molecules, thereby hindering drug release in the early stages.

#### 3.12.2. Mechanism 2: swelling-induced dense gel layer formation

Although the swelling ratio is higher at pH 5.8, excessive swelling of chitosan can lead to the formation of a dense, hydrated gel layer on the nanocomposite surface. This phenomenon, known as the “gel barrier effect,” can paradoxically slow down drug diffusion by creating a tortuous pathway for drug molecules. At pH 7.4, moderate swelling results in a more open and porous matrix structure that facilitates faster drug release. This behavior has been previously reported for chitosan-based hydrogels and nanocomposites.

#### 3.12.3. Mechanism 3: hydrophobic interactions at acidic pH

Celecoxib is a highly lipophilic drug (log *P* ≈ 3.5). At lower pH, the drug remains predominantly in its unionized, more hydrophobic form, increasing its partitioning into the hydrophobic domains of the chitosan polymer matrix. This hydrophobic retention reduces the apparent release rate despite higher water uptake. At pH 7.4, partial ionization of celecoxib increases its aqueous solubility and reduces its affinity for the hydrophobic polymer domains, thereby enhancing release.

### 3.13. Colloidal stability of the drug-loaded nanocomposite

**Fig. 20:**
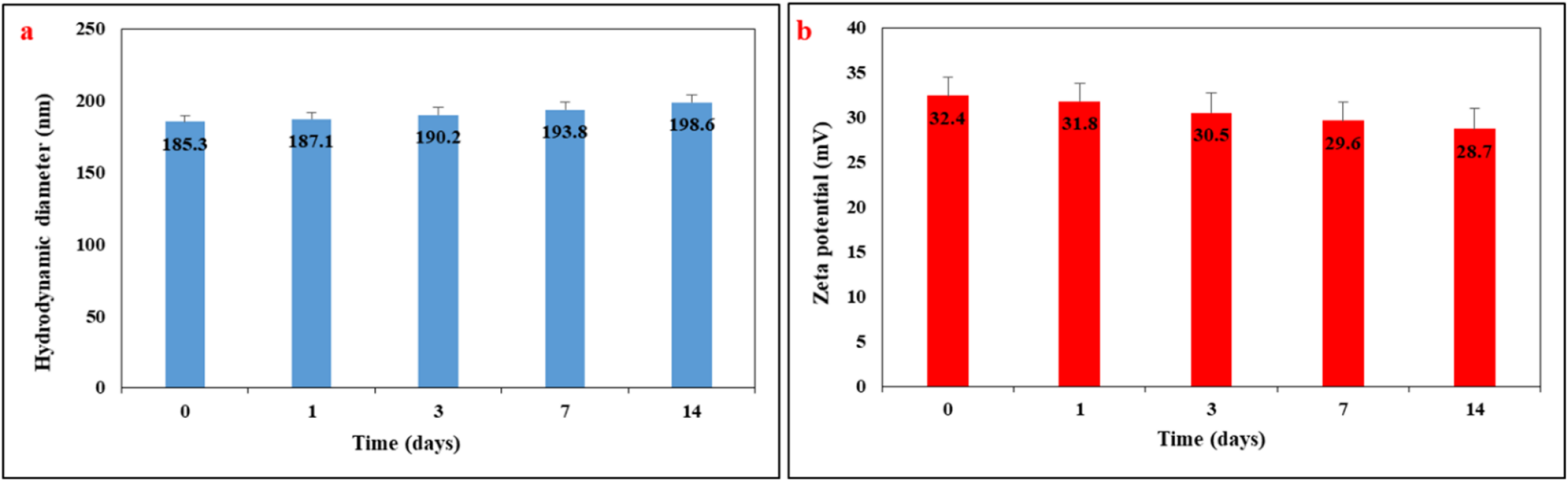
Colloidal stability of the drug-loaded LaFeO_3_/chitosan/Mg–Al LDH nanocomposite in phosphate buffer (pH 7.4, 37 °C) over 14 days: (a) hydrodynamic diameter, (b) zeta potential. Data are presented as mean ± SD (*n* = 3).

To evaluate the colloidal stability of the optimized drug-loaded LaFeO_3_/chitosan/Mg–Al LDH nanocomposite under simulated physiological conditions, the hydrodynamic diameter and zeta potential were monitored over 14 days. The nanocomposite was dispersed in phosphate buffer (pH 7.4) and maintained at 37 ± 0.5 °C. Samples were analyzed at predetermined time intervals (0, 1, 3, 7, and 14 days) using dynamic light scattering (DLS). The results are presented in Fig. 20a and b. The initial hydrodynamic diameter was 185.3 ± 4.2 nm, which increased slightly to 198.6 ± 5.1 nm after 14 days. The zeta potential decreased from an initial value of +32.4 ± 2.1 mV to +28.7 ± 2.3 mV over the same period. These minor changes indicate that the nanocomposite maintains good colloidal stability without significant aggregation or premature degradation. The consistently positive zeta potential (>+25 mV) confirms adequate electrostatic repulsion between particles, preventing agglomeration. This stability is attributed to the presence of chitosan, which acts as a steric stabilizer, and the uniform dispersion of components within the nanocomposite structure.

## 4. Conclusion

In this study, an advanced ternary nanocomposite with the structure LaFeO_3_/Mg–Al LDH/chitosan was successfully designed and synthesized as a pH-responsive controlled drug delivery platform for the anti-inflammatory drug celecoxib. Spectroscopic (FT-IR, XRD) and microscopic (SEM, EDS, and elemental mapping) characterizations confirmed that the integration of organic and inorganic components led to the formation of a stable, layered polymeric, and uniform structure with homogeneous elemental distribution. Optimization analyses using RSM and CCD revealed that the optimal conditions for drug loading were achieved at pH 9.38, a temperature 43.92 °C, and a water-to-ethanol ratio of 76.757. Under these conditions, the DLE and DLC reached 98.72% and 43.96%, respectively. The results indicated that the water-to-ethanol ratio and pH had the most significant effects on the adsorption process, while the developed statistical model demonstrated excellent predictive capability (*R*^2^ > 0.95). The drug release profile exhibited a biphasic and pH-sensitive behavior, where the release rate increased in acidic medium (pH 5.8), reaching approximately 90% release within 24 hours. This pattern reflects the nanocomposite's ability to provide an initial rapid release (burst release) for immediate therapeutic action, followed by a sustained release phase to maintain the desired therapeutic concentration. Furthermore, the swelling test showed a significant increase in swelling under acidic conditions, confirming the pH-responsive behavior of the nanocomposite. Kinetic analysis of the drug release data demonstrated that the Higuchi model (*R*^2^ = 0.9737) best described the release mechanism. Overall, the synthesized ternary nanocomposite, with its high drug loading capacity, controlled and pH-dependent release behavior, well-known biocompatibility of its primary constituents and favorable environmental stability, represents a novel and promising platform for precise drug delivery systems.

## Conflicts of interest

The authors hereby confirm that there are no known competing financial interests or personal relationships that could have appeared to exert undue influence on the work reported herein.

## Data Availability

The data that support the findings of this study are available from the corresponding author upon reasonable request.
